# Comparative transcriptome analyses of flower development in four species of *Achimenes* (Gesneriaceae)

**DOI:** 10.1186/s12864-017-3623-8

**Published:** 2017-03-20

**Authors:** Wade R. Roberts, Eric H. Roalson

**Affiliations:** 1 Molecular Plant Sciences Graduate Program, Washington State University, Pullman, WA 99164-1030 USA; 2 School of Biological Sciences, Washington State University, Pullman, WA 99164-4236 USA

**Keywords:** Comparative transcriptomics, Flower evolution, Gesneriaceae, Coexpression clustering, RNA-seq

## Abstract

**Background:**

Flowers have an amazingly diverse display of colors and shapes, and these characteristics often vary significantly among closely related species. The evolution of diverse floral form can be thought of as an adaptive response to pollination and reproduction, but it can also be seen through the lens of morphological and developmental constraints. To explore these interactions, we use RNA-seq across species and development to investigate gene expression and sequence evolution as they relate to the evolution of the diverse flowers in a group of Neotropical plants native to Mexico—magic flowers (*Achimenes*, Gesneriaceae).

**Results:**

The assembled transcriptomes contain between 29,000 and 42,000 genes expressed during development. We combine sequence orthology and coexpression clustering with analyses of protein evolution to identify candidate genes for roles in floral form evolution. Over 25% of transcripts captured were distinctive to *Achimenes* and overrepresented by genes involved in transcription factor activity. Using a model-based clustering approach we find dynamic, temporal patterns of gene expression among species. Selection tests provide evidence of positive selection in several genes with roles in pigment production, flowering time, and morphology. Combining these approaches to explore genes related to flower color and flower shape, we find distinct patterns that correspond to transitions of floral form among *Achimenes* species.

**Conclusions:**

The floral transcriptomes developed from four species of *Achimenes* provide insight into the mechanisms involved in the evolution of diverse floral form among closely related species with different pollinators. We identified several candidate genes that will serve as an important and useful resource for future research. High conservation of sequence structure, patterns of gene coexpression, and detection of positive selection acting on few genes suggests that large phenotypic differences in floral form may be caused by genetic differences in a small set of genes. Our characterized floral transcriptomes provided here should facilitate further analyses into the genomics of flower development and the mechanisms underlying the evolution of diverse flowers in *Achimenes* and other Neotropical Gesneriaceae.

**Electronic supplementary material:**

The online version of this article (doi:10.1186/s12864-017-3623-8) contains supplementary material, which is available to authorized users.

## Background

Flowers are a common way that humans connect to nature and the variety of colors and shapes remains one of the most visible and amazing products of evolution. Innovations in floral form have been proposed as one of the primary mechanisms of angiosperm diversification [1] and the phenotypic diversity of flowers is both visually striking and evolutionarily intriguing. Flower evolution is often thought about from an adaptive perspective with the evolution of floral form viewed as a function of reproductive biology or pollination biology [2]. However, developmental constraints and morphological potential can also be viewed as a function of floral organogenesis, morphology, and development rather than strictly an adaptive response [3]. In recent years, studies of flower morphology in an evolutionary and comparative context have been lifted by genetic analyses of developmental pathways underlying flower morphogenesis and biochemistry [4]. However, understanding the macroevolutionary consequences of flower modification through genetic and microevolutionary processes remains difficult. The difficulty arises from the multitude of possible genetic changes available to produce these phenotypic adaptations. Combining the power of transcriptome sequencing with comparative floral morphology allows for the exploration of the possible evolutionary genetic mechanisms involved in flower development and diversification.

**Fig. 1:**
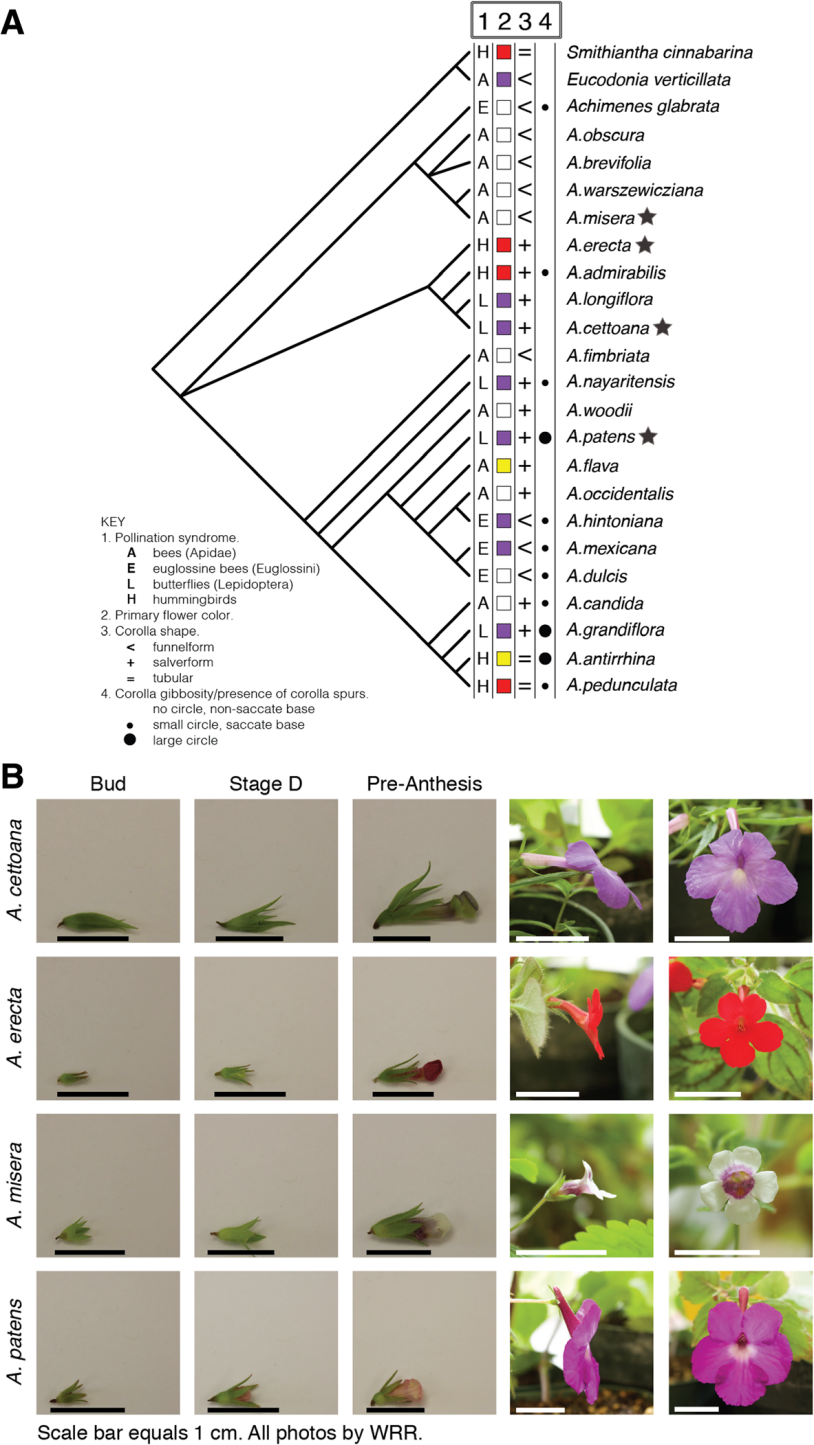
Phylogenetic relationships (modified from Roalson et al. [10]) and flower developmental stages sampled in *Achimenes.* **a** Floral morphological characters of interest are mapped onto the tips of the cladogram, including: pollination syndrome, primary flower color, corolla shape, and corolla gibbosity/presence of petal spurs. Four species were sampled for this study from across the genus and are indicated by a star. **b** The time-points sampled were: Bud, Stage D, and Pre-Anthesis flower buds. Bud stage was defined as pigmentation is largely absent and cells are beginning to elongate. Stage D was defined as pigmentation beginning to accumulate and the corolla begins to elongate. Pre-Anthesis stage was defined as flowers are nearly fully pigmented, the final size and shape of the flower has been determined, and the petal spur has developed from the corolla tube (as in *A. patens*). Scale bar equals 1 cm. All photos provided by W.R.R

We provide a first characterization of the floral transcriptomes in four species of magic flowers, *Achimenes*. This small genus of ~26 species is a member of the African violet family (Gesneriaceae), a large family distributed in the New World and Old World tropics. The family is renowned for its enormous diversity in habit, desiccation tolerance, leaf morphology, and, particularly, floral form [5–7]. Flower shape, color, and presentation are hypothesized to be important for diversification and speciation events in the family [7–11]. Convergence in floral form is found across the family as well as in individual genera and is likely tied to pollinator preferences and pollinator availability [7, 11]. In *Achimenes*, floral form appears to be quite variable among closely related species and similar corolla shapes and colors are found among species that occur in different clades [10] (Fig. 1). Multiple derivations of flower shape, color, and the presence of a petal spur appear across the genus [10] (Fig. 1). Populations of *Achimenes* are largely concentrated in central Mexico south to Costa Rica, with some populations existing in the Caribbean. General distributions of many closely related species often overlap with many populations found growing in the same habitat and elevation ranges [12]. Pollinator studies have been limited with observations recorded for only four species of *Achimenes* [13]. The major pollinator observed for each of the four *Achimenes* species corresponds tightly with the hypothesized pollination syndrome that was identified using combinations of floral traits thought to be important for pollinator attraction, such as color, shape, size, and orientation of the open flower [10]. The young age of the genus (~12 Ma) [7], coupled with a large number of shifts in flower shape, color, and pollination syndrome [10], makes *Achimenes* an ideal lineage to begin understanding the ecological, evolutionary, and molecular forces contributing to speciation and diversification of floral form.

Here, we present *de novo* floral transcriptome assemblies of four species of Neotropical *Achimenes* (Gesneriaceae) that vary in floral form, pigmentation patterns, and pollination syndrome. Diversity of flower shape and color among sister species in *Achimenes* present intriguing questions about the ecological and genetic forces contributing to these phenotypic divergences. We sampled flowers in three developmental stages from *A. cettoana, A. erecta, A. misera,* and *A. patens*. This sampling strategy allows inter- and intraspecies comparisons of gene expression during development and comparisons of sequence structure in order to begin investigating evolutionary and developmental mechanisms contributing to speciation and diversification. Utilizing high-throughput technologies has allowed researchers in both animal [14, 15] and plant [16–18] systems to sequence entire genomes, transcriptomes, and proteomes in order to understand fine-scale patterns of genetics and evolution. Our study takes advantage of these genomic approaches and provides resources that will serve as the basis for future studies into flower development, evolution, and plant-pollinator interactions.

Comparative transcriptomic studies in plants have seen an increasing publication rate in recent years as sequencing technologies keep increasing data output for lower cost. Many studies have taken a focused look at comparing developmental stages in a single species across different tissues [19–21], comparing gene expression in different organs [22, 23], or simply to generate preliminary genomic data that will guide more detailed studies [24–27]. Evolutionary questions have also been investigated using genome-wide expression data in plants, such as the evolution of gene expression patterns [16], parasitism [18], self-fertilization [28], or mass flowering [29]. Our study aims to bridge the gap between these different areas. We took a developmental approach by sampling several stages of flower development and an evolutionary approach by comparing transcriptome data across multiple species. This evolutionary-developmental approach to comparative transcriptomics presents a novel way to investigate the patterns and processes of flower diversification at the genomic level. This study provides annotated reference transcriptomes for four species of *Achimenes* and uses them for analyses of sequence orthology, coexpression clustering of genes during development, and selection tests to identify protein sites undergoing positive selection. We also use data from the transcriptomes to begin investigating the genetics of flower color, particularly the production of anthocyanin pigments. It is our goal that the resources and results provided herein will serve as the basis for future studies. This study is among the first explorations of Neotropical Gesneriaceae flower transcriptomes using large-scale sequencing, and the results described here may serve to guide further gene expression and functional genomic studies in *Achimenes* and other members of the Gesneriaceae.

## Results

### Assembly of high-quality achimenes floral transcriptomes

Sequencing the floral transcriptomes from three developmental stages in four species of *Achimenes* yielded over 270 million reads (Table 1). Each species had between 63 and 72 million paired-end reads sequenced (Table 1).

**Table 1:** Sequencing and summary statistics for *Achimenes* reference floral transcriptome assemblies and annotation

	*A. cettoana*	*A. erecta*	*A. misera*	*A. patens*
A. Sequencing				
Total reads	67,428,998	63,582,836	69,588,964	71,960,488
Bud	21,112,016	18,680,312	24,016,214	22,585,994
Stage D	22,382,106	24,084,300	19,391,388	28,579,042
Pre-Anthesis	23,934,876	20,818,224	26,181,362	20,795,452
Total length (bp)	6,742,899,800	6,358,283,600	6,958,896,400	7,196,048,800
B. Final merged assembly				
Primary transcripts	29,065	41,381	41,285	37,898
Alternate transcripts	23,332	94,172	105,442	65,115
N50	2,113	2,061	1,990	2,109
Mean length (bp)	1,417	1,268	1,260	1,304
Total bases, Primary set	41,202,771	52,511,722	52,038,201	49,447,956

Trinity assemblies using a *k*-mer size of 25 produced between 139,806 (*A. cettoana*) and 199,502 (*A. erecta*) contigs for each reference transcriptome (Additional file 1). These assembled contigs had N50 values between 1444 (*A. misera*) and 1794 (*A. cettoana*) bps, with mean lengths between 868 (*A. misera*) and 1027 (*A. cettoana*) bps (Additional file 1). Velvet and Oases assemblies were also performed using a range of *k*-mer sizes from 25 to 75 (Additional file 1). Generally, these assemblies produced higher numbers of contigs, with higher N50 values, and higher mean values than the Trinity assemblies (Additional file 1). The number of contigs ranged from 46,189 in *A. cettoana* using a *k-*mer size of 75 to 247,516 in *A. erecta* using a *k*-mer size of 35 (Additional file 1). N50 values were also showed some variation consistent with larger *k*-mers producing lower values (1,385 in *A. misera*) and smaller *k*-mers producing higher values (2,334 in *A. erecta*; Additional file 1). Assemblies for *A. cettoana* always produced far fewer contigs than the other species (e.g., using Velvet/Oases, 126,317 in *A. cettoana* versus 247,516 in *A. erecta*, see Additional file 1). The number of contigs assembled does not appear to negatively affect other assembly metrics; the mean length and N50 values were similar across all species assemblies (Additional file 1).

Merging the separate *de novo* assemblies reduced redundancy and provided useful sets of contigs for further analyses (Table 1; Additional file 1). Between 29,065 and 41,381 primary transcripts were obtained with N50 lengths between 1,990 and 2,113 bps (Table 1). The merging process also provided between 23,332 and 105,442 alternate transcripts, which are composed of possible isoforms (Table 1; Additional file 1).

### Functional annotation and classification

The primary floral transcriptomes of *A. cettoana, A. erecta, A. misera*, and *A. patens* were annotated by BLASTx searches against the SwissProt [30] and the NCBI non-redundant (Nr) protein database [31]. For *A. cettoana*, 18,364 (63.18%) sequences had hits in the SwissProt database; *A. erecta*, 23,534 (56.87%) sequences had hits; *A. misera*, 23,120 (56.00%) sequences had hits; and *A. patens,* 20,838 (54.98%) sequences had hits (Table 2). The numbers of sequences with at least 75% coverage by their best protein hits were 10,281 (35.37%), 12,372 (29.90%), 11,420 (27.66%), and 11,097 (29.28%), for each transcriptome respectively. Against the Nr database, *A. cettoana* had 23,012 (79.17%) sequences with hits; *A. erecta* had 29,794 (72.00%) sequences with hits; *A. misera* had 29,783 (72.14%) sequences with hits; and *A. patens* had 26,776 (70.65%) sequences with hits (Table 2). Additionally, we performed BLASTn searches against a collection of *Arabidopsis thaliana* long non-coding RNA (lncRNA) sequences acquired from the Plant Non-coding RNA Database [32]. Against this set, *A. cettoana* had 76 (0.0026%) sequences with hits; *A. erecta* had 96 (0.0023%) sequences with hits; *A. misera* had 85 (0.0021%) sequences with hits; and *A. patens* had 117 (0.0031%) sequences with hits (Table 2). Non-coding ribosomal RNAs and tRNAs formed a small number of the total contigs (Additional file 2).

**Table 2:** Overview of BLAST hits to primary transcript set and functional annotation output of the four reference transcriptomes

	*A. cettoana*	*A. erecta*	*A. misera*	*A. patens*
SwissProt	18,365 (63.18%)	23,534 (56.78%)	23,120 (56.00%)	20,838 (54.98%)
Nr	23,012 (79.17%)	29,794 (72.00%)	29,783 (72.14%)	26,776 (70.65%)
PNRD	76 (0.0026%)	96 (0.0023%)	85 (0.0021%)	117 (0.0031%)
GO	11,826 (40.69%)	14,996 (36.24%)	14,683 (35.56%)	13,179 (34.78%)

*Abbreviations: GO* gene ontology, *Nr* NCBI non-redundant protein database, *PNRD* plant non-coding RNA database

The sequences with matches in the SwissProt [30] or Nr [31] databases were further annotated with Gene Ontology (GO) terms [33] based on the SwissProt database, InterProScan [34], and ANNEX augmentation [35]. GO terms were assigned to 11,826 (40.69%) transcripts in *A. cettoana*, 14,996 (36.24%) transcripts in *A. erecta*, 14,683 (35.56%) transcripts in *A. misera*, and 13,179 (34.78%) in *A. patens* (Table 2)*.* Numbers and proportions of sequences attributed to level 2 GO for Biological Process (BP), Cellular Component (CC), and Molecular Function (MF) type terms were qualitatively similar with slight variations likely due to numbers of transcripts assembled for each species (Additional file 3). Representation was qualitatively very similar between the four species, with all level 2 GO categories exhibiting no significant differences across species even after accounting for the effects of multiple testing (*Χ* ^*2*^ ≥ 1.65, FDR-corrected *p-value* $\ge 0.9\overline{9}$, *α* = 0.001). We looked at further GO levels (level 3, level 4, etc.) and found similar composition of category assignment for each transcriptome.

**Fig. 2:**
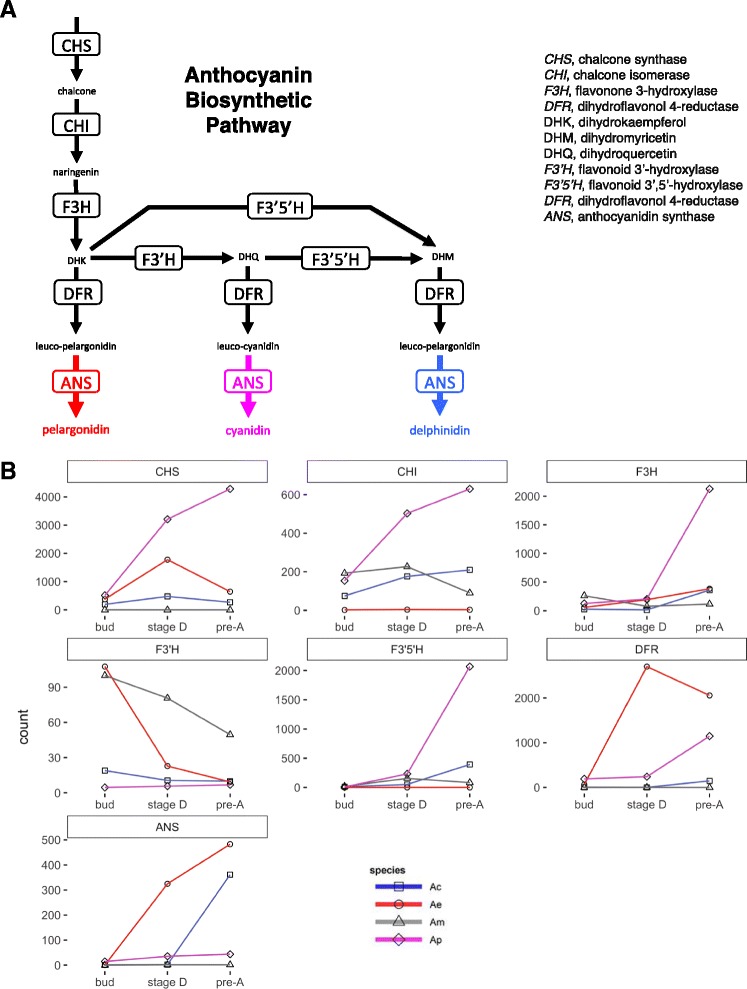
Expression estimates for core enzymes of the anthocyanin biosynthetic pathway in *Achimenes*. LEGEND: **a**. Schematic outline of the core anthocyanin biosynthetic pathway in plants. **b**. Expression (TPM) for the core enzymes of the anthocyanin biosynthetic pathway during flower development in *Achimenes*. Expression for *A. cettoana*, *A. erecta*, *A. misera*, and *A. patens* are in blue, red, grey, and pink lines, respectively

Core enzymes of the anthocyanin biosynthetic pathway (ABP) were identified using HMMER [36] against homologs downloaded from GenBank (Additional file 4). The HMMER searches identified 224 proteins with similarity to anthocyanidin synthase (*ANS*, Additional file 5), 122 proteins with similarity to dihydroflavonol 4-reductase (*DFR*, Additional file 6), and 730 proteins with similarity to both *F3′H* (flavonoid 3′-hydroxylase, Additional file 7) and *F3′5′H* (flavonoid 3′,5′-hydroxylase, Additional file 7). These large groups of proteins represent putative gene families for each of these enzymes. Aligning the sequences of these proteins with the sequences of known proteins from other studies and constructing neighbor-joining trees allowed us to identify putative proteins from *Achimenes* involved in the ABP. We identified single copies of *ANS* (Additional file 5), *DFR* (Additional file 6; Additional file 8), *F3′H* (Additional file 7; Additional file 8), and *F3′5′H* (Additional file 7; Additional file 8) in each transcriptome, with the exception of *A. misera* where 6 copies of *F3′5′H* were identified (Additional file 7). Five of the six *A. misera* copies have very low normalized expression estimates and may represent genes that are expressed at too low of level to be detected at the current sequencing depth or may be artifacts of our assembly process. Expression estimates for each of the identified single copy enzymes generally increases from B to A stages (Fig. 2) as pigments accumulate in the floral tissue.

**Fig. 3:**
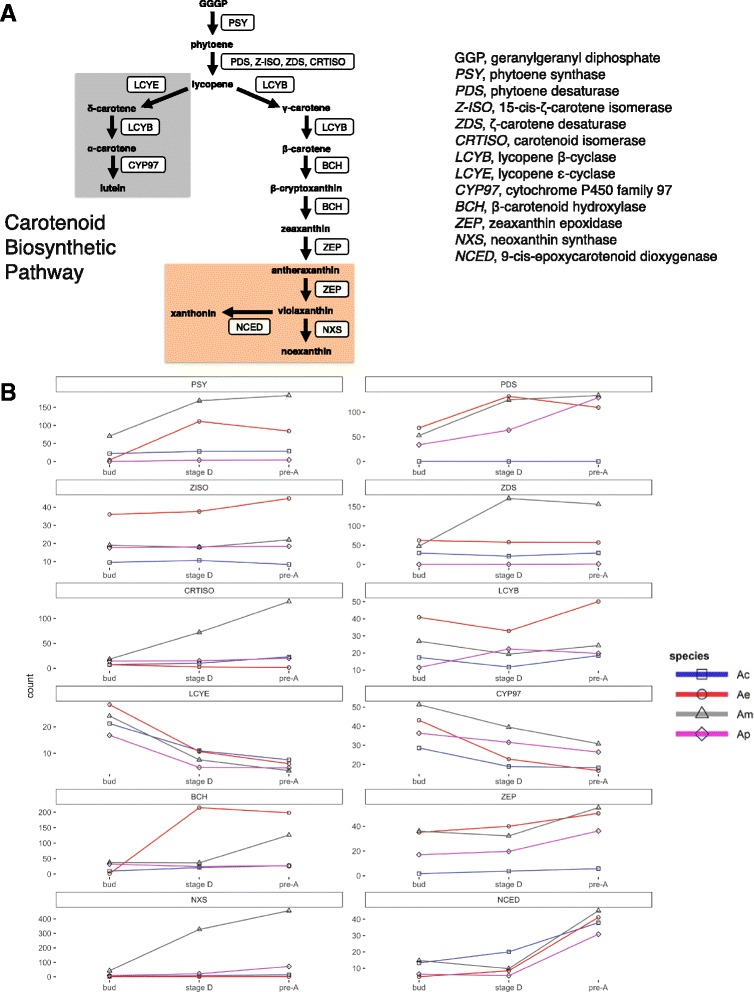
Expression estimates for core enzymes of the plant carotenoid biosynthetic pathway in *Achimenes*. **a** Schematic outline of the plant carotenoid biosynthetic pathway. The enzymes are shown in boxes to the side of the arrows. *Grey* and *orange boxes* indicate the α-carotene and β-carotene branches, respectively. **b** Expression (TPM) for the core enzymes of the carotenoid biosynthetic pathway during flower development in *Achimenes.* Expression for *A. cettoana*, *A. erecta*, *A. misera*, and *A. patens* are in *blue*, *red*, *grey*, and *pink lines*, respectively

Putative enzymes of the carotenoid biosynthetic pathway (CBP) were identified from each *Achimenes* transcriptome using BLASTx. Both bit scores and E-values were used to identify best-hit transcripts. Using homologs from *Arabidopsis* as query, there were 12 proteins identified to be involved in carotenoid biosynthesis (Fig. 3). Proteins identified included ones belonging to both the α-carotene and β-carotene branches (Fig. 3).

Proteins related to flower development were additionally identified from each *Achimenes* transcriptome using BLASTx. We used both bit scores and E-values to identify putative proteins. Using homologs from *Arabidopsis* as query, there were 101 putative proteins identified that may be involved in flower development (Additional file 9). These included proteins involved in flowering transition, organ development, and floral repression (Additional file 9, Additional file 10). Among the proteins identified were A-, B-, C-, and E-class MADS-box genes, members of the AP2/ERF family, numerous homeobox genes, and many others (Additional file 9, Additional file 10). Each of these proteins has a distinct expression domain during development and may be expressed in floral organs (sepals, petals, etc.), in the floral meristem, or in the inflorescence (Additional file 9, Additional file 10).

**Fig. 4:**
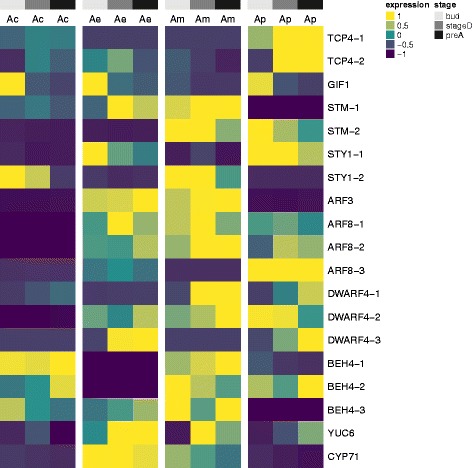
Expression of putative genes involved in petal spur development in *Achimenes.* Heatmap of scaled expression estimates for 10 *Achimenes* homologs of *Aquilegia* genes hypothesized to be important for petal spur development according to [37]. Rows and columns are not clustered

Several genes involved in cell proliferation and hormone signaling were recently identified to be important for petal spur development in *Aquilegia* [37]. We identified homologs of these genes from each transcriptome using both bit scores and E-values to select likely candidate transcripts. The *Achimenes* transcripts identified include homologs of *TCP4* and *GIF1*, both involved in cell division control (Fig. 4). *TCP4* distinctly shows very high expression in *A. patens* and not the other *Achimenes* species, a similar pattern to that observed in *Aquilegia* [37] (Fig. 4). Other genes identified include *STM* involved in meristem indeterminacy [38], *STY1* that regulates auxin biosynthesis [39], *ARF3* and *ARF8* that are auxin response factors, *YUC6* and *CYP71* both involved in auxin biosynthesis, and *DWARF4* and *BEH4* that function in the brassinosteroid pathway [40, 41] (Fig. 4).

Lastly, we identified candidate R2R3-Myb transcription factors that may be involved in regulating anthocyanin and carotenoid biosynthesis in flowers. Using HMM profiles built from R2R3-Mybs shown to be involved in these pathways, we identified several candidate proteins. There are 8 *Achimenes* sequences identified that are closely related to R2R3-Mybs from *Erythranthe* and *Antirrhinum* that regulation floral anthocyanin production (Additional file 11). Nine *Achimenes* sequences were identified and related to an R2R3-Myb transcription factor in *Erythranthe* that regulated floral carotenoid production (Additional file 11).

### Core, shared, and unique genes

**Fig. 5:**
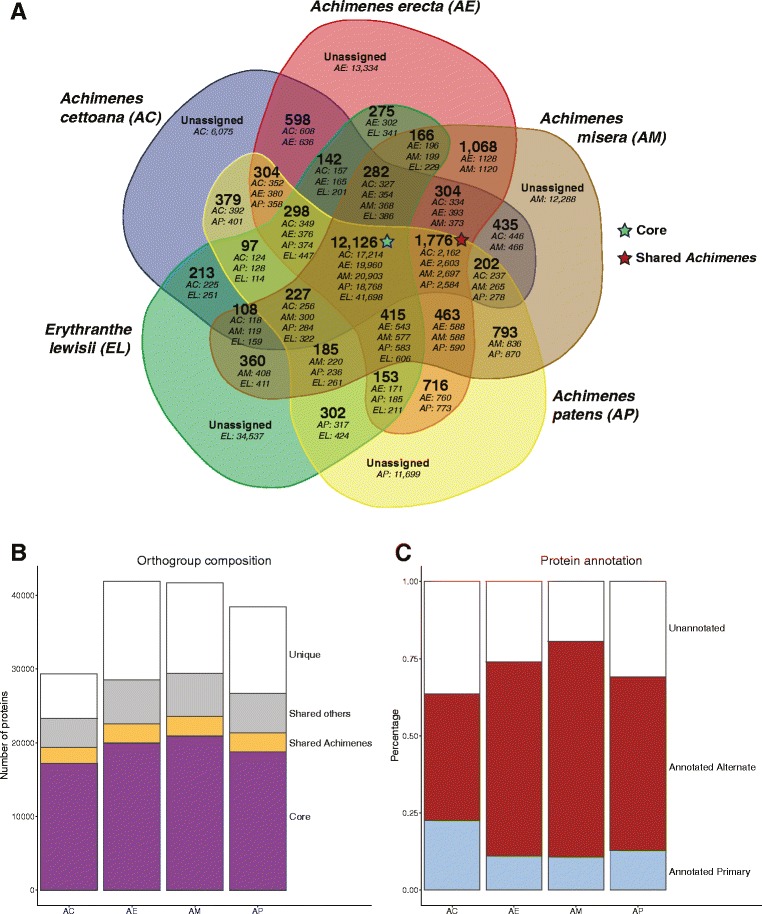
Sequence orthology and the proportion of annotated sequences in the *Achimenes* transcriptomes. **a** Venn diagram showing the number of shared or unique genes (in italics) and gene clusters (*bold*) among the five *Achimenes* species and *Erythranthe* as classified by the OrthoFinder program. “Core” and “Shared *Achimenes*” orthogroups are indicated with *blue* and *red stars*, respectively. Abbreviations: AC, *Achimenes cettoana*; AE, *Achimenes erecta*; AM, *Achimenes misera*; AP, *Achimenes patens*; EL, *Erythranthe lewisii*. **b** Proportion of the transcripts that comprised core, shared *Achimenes,* shared others, and unique genes. “Core” orthogroups were common to all four *Achimenes* and *Erythranthe.* “Shared *Achimenes*” are orthogroups that contain sequences from all four *Achimenes* species. “Shared other” are orthogroups present in two or three of the four *Achimenes* species. “Unique” genes are genes that are only present in one species and were unassigned to a specific orthogroup. **c** Proportion of annotated and non-annotated genes in the primary and alternate transcriptomes

We found a set of gene clusters that were common to all four *Achimenes* species and the outgroup *Erythranthe lewisii* (collectively termed the “Core transcriptome”). This core set of proteins consisted of 12,126 gene clusters (Fig. 5), which comprised 59%, 48%, 50%, and 49% of the total predicted proteins in *A. cettoana, A. erecta, A. misera,* and *A. patens*, respectively (Fig. 5). There were an additional 1,776 gene clusters (Fig. 5) that were unique and shared among all four gesneriad species (“Shared *Achimenes*”). These clusters comprised 7.4%, 6.2%, 6.5%, and 6.7% of the total predicted proteins, respectively (Fig. 5). In addition to the shared clusters within *Achimenes,* each species also contained unique protein sequences (unassigned to any cluster) that were not found in any of the other five transcriptomes; these unique sequences comprised 21–32% of the transcriptomes (Fig. 5). Approximately 14% of the transcriptomes were comprised of protein orthogroups shared between at least two of the five species (“Shared others”, Fig. 5).

Among the 12,126 orthogroups that were shared by all five species in the five-way comparison, there were 78 GO terms significantly enriched (FDR-corrected *p*-value < 0.05). As expected, most of these terms were related to primary metabolism, cellular components and structure, signaling, reproduction, and response to stimulus, among many others (Additional file 12). Within the protein clusters that were shared among all four *Achimenes* species (“Shared *Achimenes*”), 27 GO terms were significantly overrepresented (FDR-corrected *p*-value <0.05) in all species (Additional file 13). When comparing protein sequences that each species contributed to the “Shared *Achimenes*” orthogroup, there were 7 overrepresented GO terms identified in all four species individually. Interestingly, each of these terms were involved in DNA binding, including chromatin binding and transcription factor activity (Table 3). Among the sequences that were unassigned to any clusters, there were some differences in the number and type of GO terms that were significantly over- or underrepresented in each species, with 4 terms identified in *A. patens* and 26 terms identified in *A. erecta.* (Additional file 14).

**Table 3:** Gene ontology terms overrepresented in the “Shared *Achimenes*” orthogroups

Term	Description	Type	FDR	Single-test *p*-value	Number in test group	Number in reference group
GO:0003676	Nucleic acid binding	MF	4.20e-46	2.30e-48	977	7373
GO:0003677	DNA binding	MF	2.10e-44	2.30e-46	612	3951
GO:0003682	Chromatin binding	MF	3.10e-28	6.80e-30	157	619
GO:0044877	Macromolecular complex binding	MF	3.10e-28	6.80e-30	157	619
GO:0003700	Transcription factor activity, sequence-specific DNA binding	MF	6.30e-20	2.00e-21	200	1112
GO:0001071	Nucleic acid binding transcription factor activity	MF	6.30e-20	2.00e-21	200	1112
GO:1901363	Heterocyclic compound binding	MF	2.70e-08	1.20e-09	1517	16173
GO:0097159	Organic cyclic compound binding	MF	2.70e-08	1.20e-09	1517	16173
GO:0005618	Cell wall	CC	3.10e-06	1.50e-07	43	199
GO:0030312	External encapsulating structure	CC	4.50e-06	2.40e-07	43	203
GO:0005488	Binding	MF	1.30e-04	8.00e-06	2737	31646
GO:0090304	Nucleic acid metabolic process	BP	2.70e-04	1.90e-05	145	1210
GO:0006259	DNA metabolic process	BP	2.70e-04	1.90e-05	145	1210
GO:0030246	Carbohydrate binding	MF	2.10e-03	1.60e-04	63	456
GO:0019825	Oxygen binding	MF	5.60e-03	4.60e-04	5	5
GO:0071944	Cell periphery	CC	5.60e-03	4.90e-04	58	430
GO:0009653	Anatomical structure morphogenesis	BP	7.00e-03	6.50e-04	7	14
GO:0015979	Photosynthesis	BP	9.40e-03	9.20e-04	35	229
GO:0006725	Cellular aromatic compound metabolic process	BP	1.90e-02	2.30e-03	590	6399
GO:1901360	Organic cyclic compound metabolic process	BP	1.90e-02	2.30e-03	590	6399
GO:0046483	Heterocycle metabolic process	BP	1.90e-02	2.30e-03	590	6399
GO:0006139	Nucleobase-containing compound metabolic process	BP	1.90e-02	2.30e-03	590	6399
GO:0005576	Extracellular region	CC	2.10e-02	2.60e-03	31	209
GO:0005634	Nucleus	CC	2.80e-02	3.70e-03	236	2391
GO:0009607	Response to biotic stimulus	BP	3.80e-02	5.20e-03	19	113
GO:0005615	Extracellular space	CC	4.20e-02	6.20e-03	6	17
GO:0044421	Extracellular region part	CC	4.20e-02	6.20e-03	6	17

*Abbreviations: BP* biological process, *CC* cellular component, *FDR* false discovery rate corrected *p*-value, *MF* molecular function

### Quantifying expression and coexpression clustering

We estimated gene expression by mapping RNA-seq reads from each developmental stage (B, Immature Bud; D, Stage D; A, Pre-Anthesis) back to the respective reference ‘primary’ transcriptome using bowtie [42] and RSEM [43]. In each of the four species (*A. cettoana, A. erecta, A. misera,* and *A. patens*), the mapping rate averaged 93.59%, 93.39%, 95.1%, and 92.07%, respectively. Additionally, mapping reads from one species onto another species reference produced successful mapping rates of >85%.

**Fig. 6:**
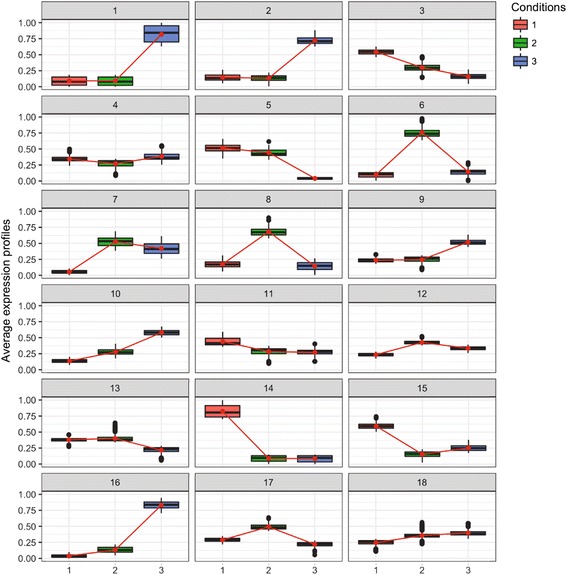
Coexpression cluster profiles of *Achimenes cettoana* transcripts using Poisson mixture models. Thirty-four coexpression clusters were determined for *A. cettoana* with Poisson mixture models using slope heuristics as implemented in [44]. Clusters 1 to 18 are presented here to provide an example of the dynamic patterns of gene coexpression seen during flower development in *A. cettoana.* The full figure showing all 34 clusters is included in Additional file 15. Boxplots indicate average gene expression profiles for each cluster. Conditions refer to the sampled stages of flower development: 1, Bud stage; 2, Stage D; and 3, Pre-Anthesis stage

Over 5 independent runs, we used HTSCluster [44] and the EM algorithm [45] to fit a sequence of Poisson mixture models with *K =* 1, 2, …, 60 clusters for the expression estimates of each reference transcriptome. Using slope heuristics (Djump, dimension jump; DDSE, data driven slope estimation) [46], the number of clusters was determined to be *K* = 34, 30, 29, 25 for the *A. cettoana, A. erecta, A. misera,* and *A. patens* expression estimates, respectively. Visualization of the clustering displays numerous clusters with very high or very low expression levels during specific stages in development and also many clusters where expression is not qualitatively different between the three stages (Fig. 6; Additional file 15). Visualization of the maximum conditional probabilities of cluster membership for each species indicates confidence in cluster assignment (Additional file 16), particularly among clusters that have distinct high or low expression during a single developmental stage (Fig. 6; Additional file 15, Additional file 16). Examining what, if any, GO terms may be over- or underrepresented in specific coexpression clusters may be useful to determine any temporal patterns of gene expression during flower development. In *A. cettoana*, 22 of 34 (65%) clusters had significantly over-enriched GO terms associated. Likewise, *A. erecta* had 23 of 30 (77%), *A. misera* had 21 of 29 (72%), and *A. patens* had 21 of 25 (84%) clusters with significantly over-enriched GO terms (Additional file 17).

GO term enrichment tests were performed for each cluster to identify general patterns of gene coexpression (Additional file 18). Trends in gene coexpression were apparent and what we expect for developing flowers. For instance, genes involved in photosynthesis tended to have higher expression in the B stage, while genes involved in primary metabolism and biosynthetic processes tended to be enriched in clusters without qualitative differences between stages (Additional file 18). While considering broad-scale patterns of gene categories that tend to be coexpressed together provided important results, we additionally wanted to investigate which clusters contained genes involved in flower shape and pigment production.

Many members of the ABP were coexpressed together (Additional file 19). In three species (*A. cettoana*, *A. erecta*, and *A. patens*), several of the downstream enzymes were found in the same coexpression cluster, including *F3H*, *F3′H*, *F3′5′H*, *DFR*, and *ANS* (Additional file 19). In *A. misera*, all enzymes were put into different coexpression clusters with the exception of *CHI* and *F3′H* (Additional file 19). Several of the candidate R2R3-Mybs identified were also coexpressed with enzymes of the ABP (Additional file 19). One R2R3-Myb was coexpressed in *A. cettoana* with *F3′5′H*; one was coexpressed in *A. misera* with *ANS*; and one was coexpressed in *A. patens* with *F3H*, *F3′5′H*, and *ANS* (Additional file 19).

There were very few enzymes of the CBP that were found in the same coexpression cluster (Additional file 19). The downstream enzymes of the β-carotene branch tended to be found in the same coexpression cluster in some species, particularly *BCH*, *ZEP*, *NXS*, and *NCED* (Additional file 19). Of the 9 candidate R2R3-Mybs identified, only one in *A. erecta* was coexpressed with any of the CBP enzymes, namely *CYP97* (Additional file 19).

The genes identified to be involved in flower development did not show any clear coexpression patterns. For instance, genes that are involved in petal or carpel development are found across many different clusters likely due to very different temporal patterns of gene expression (Additional file 9, Additional file 10). Likewise, the candidate genes we looked at for involvement in petal spur development show very few coexpression patterns (Additional file 19, Additional file 10). Some transcripts of particular genes were coexpressed together, *TCP4* in *A. patens* for example, while most others were found in different coexpression clusters (Additional file 19).

### Detecting proteins under selection

As detection of positive selection requires a minimum of five species to obtain reliable estimates [47], orthogroups from the five-way analysis were stringently filtered. These filtering steps provided 2,930 orthogroups, containing 26,141 total sequences, for selection analyses. Sequence alignments were visually inspected to identify spurious alignments that could produce false positives in our selection analyses. After inspection, no clusters were removed from the subsequent analyses. Likelihood ratio tests comparing four models (M1a vs. M2a, M7 vs. M8) [48, 49] were employed to identify proteins and amino acids within those proteins potentially displaying signatures of selection. Comparison of M1a versus M2a (m12) identified 339 orthogroups containing proteins with signatures of selection, while M7 versus M8 comparisons (m78) identified 642 orthogroups (FDR-corrected *p*-values ≤ 0.05). Three hundred thirty-five orthogroups were identified by both m12 and m78 comparisons. The numbers of proteins identified in m12 were 64, 80, 68, and 76 for *A. cettoana, A. erecta, A. misera,* and *A. patens*, respectively (Additional file 20). In the m78 comparison, there were 125, 144, 133, and 143 proteins identified, respectively (Additional file 21).

Enrichment tests did not show any GO terms significantly over- or underrepresented in the list of proteins with sites undergoing positive selection. Comparisons were made both for the combined set of proteins, as well as protein sets for each individual species (including *Erythranthe*). However, several GO categories of interest to the current study were found in the protein set, including terms including flower development, anatomical structure morphogenesis, anthocyanin pigmentation, and transcription factor activity (Table 4).

**Table 4:** Genes under positive selection in *Achimenes* and *Erythranthe* related to flower development and pigmentation

Gene	Description	UniProd ID	Species	Test
5MAT1	Malonyl-coenzyme:anthocyanin 5-O-glucoside-6”’-O-malonyltransferase	Q8W1W9	Ac	m12,m78
SFH13	Phosphatidylinositol/phosphatidylcholine transfer protein SFH13	Q501H5	Ac	m78
UBC28	Ubiquitin-conjugating enzyme E2 28	Q94F47	Ac	m78
Y5241	Probable receptor-like protein kinase at5g24010	Q9FLW0	Ac	m12
LECRK91	L-type lectin-domain containing receptor kinase	Q9LXA5	Ac	m12
GAUT14	Galactouronosyltransferase 14	Q8GWT1	Ac	m12
ABCB19	ABC transporter B family member 19	Q9LJX0	Ae	m12
ACR4	ACT domain-containing protein ACR4	Q8LJW3	Ae	m12
CYP90A1	Cytochrome P450 90A1	Q42569	Ae	m12
DCR	BAHD acyltransferase DCR	Q9FF86	Ae	m12
DFRA	Dihydroflavonol 4-reductase	P51102	Ae	m78
MAP70.2	Microtubule-associated protein 70-2	Q8L7S4	Ae	m12
TA14B	Transcription initiation factor TFIID subunit 14b	Q9FH40	Ae	m12
TKPR1	Tetraketide alpha-pyrone reductase 1	Q500U8	Ae	m12
GPPL2	Haloacid dehalogenase-like hydrolase domain-containing protein at3g48420	Q94K71	Ae	m12
HAT	Zinc finger bed domain-containing protein DAYSLEEPER	Q9M2N5	Ae	m12
KDSB	3-deoxy-manno-octulosonate	Q9C920	Ae	m12
BIG1	Brefeldin A-inhibited guanine nucleotide-exchange protein 1	FAJSZ5	Ae	m12
PRMT13	Probable histone-arginine methyltransferase 1.3	Q84W92	Am	m12
ATX1	Copper transport protein ATX1	Q94BT9	Am	m12
CYP71A1	Cytochrome P450 71A1	P24465	Am	m12
FLXL1	Protein FLX-like 1	Q93V84	Am	m12,m78
FRI	Protein FRIGIDA	P0DH90	Am	m12,m78
DTX41	Protein DETOXIFICATION 41	Q9LYT3	Am	m12
Y1301	BTB/POZ domain-containing protein at1g03010	Q9SA69	Am	m12
AKR2A	Ankyrin repeat domain-containing protein 2A	Q9SAR5	Ap	m78
CAF2M	CRS2-associated factor 2	Q9FFU1	Ap	m12
CKB2	Casein kinase II subunit beta-2	P40229	Ap	m12
GN	ARF guanine-nucleotide exchange factor GNOM	Q42510	Ap	m12
WNK1	Serine/threonine-protein kinase WNK1	Q9CAV6	Ap	m12
HDA19	Histone deactylase 19	O22446	Ap	m12
PRXQ	Peroxiredoxin chloroplastic	Q6UBI3	Ap	m12
CPK13	Calcium-dependent protein kinase 13	Q8W4I7	Ap	m12
LOL2	Protein LOL2	O65426	Ap	m12
EXO70A1	Exocyst complex component EXO70A1	Q9LZD3	Ap	m12
PRMT11	Protein arginine N-methyltransferase 1.1	Q9SU94	El	m12
BLH8	BEL1-like homeodomain protein 8	Q9SJJ3	El	m12
GDL15	GDSL esterase/lipase at1g29670	Q9C7N4	El	m12
GK-2	Guanylate kinase 2	Q9M682	El	m12
CUT1	3-ketoacyl-CoA synthase 6	Q9XF43	El	m12
AATL1	Lysine histidine transporter-like 8	Q9SX98	El	m12,m78
MAA3	Probable helicase MAGATAMA 3	B6SFA4	El	m12,m78
RH27	DEAD-box ATP-dependent RNA helicase 27	Q9SB89	El	m12
SOBIR1	Leucine-rich repeat receptor-like serine/threonine/tyrosine-protein kinase SOBIR1	Q9SKB2	El	m12
BRM	ATP-dependent helicase BRM	Q6EVK6	El	m12
AGO2	Protein argonaute 2	Q9SHF3	El	m12
ARID5	AT-rich interactive domain-containing protein 5	Q0WNR6	El	m12

*Abbreviations: Ac Achimenes cettoana*, *Ae Achimenes erecta*, *Am Achimenes misera*, *Ap Achimenes patens*, *El Erythranthe lewisii*, *m12* PAML model comparison m1a vs. m2a, *m78* PAML model comparison m7 vs. m8

## Discussion

This study is among the first to employ RNA sequencing for comparative studies both between species and between developmental stages in flowering plants [16, 18]. This study is also among the first to characterize and annotate floral transcriptomes in Neotropical Gesneriaceae, a lineage well known for diverse and colorful flowers [50]. *Achimenes* offers a unique opportunity to study the genomics of flower diversification in a comparative context because very closely related species display an extraordinary range of morphological diversity likely tied to pollinator preferences and shifting patterns of gene expression. Rather than using a candidate-gene approach to understand patterns of speciation and diversification, we utilize high-throughput sequencing to begin searching for the potential pathways involved. We assembled between 29,000 and 42,000 putatively unique primary and alternate transcripts for four species of *Achimenes* that display many of the most common floral forms found in the genus. Orthogroup detection among *Achimenes* and against an *Erythranthe* corolla transcriptome revealed numerous conserved and distinct transcript clusters expressed among species (Fig. 5). Coexpression clustering revealed distinct patterns of gene expression in different stages of development (Fig. 6; Additional file 15). Assessing protein sequences for signatures of positive selection revealed numerous protein sites under selection in proteins involved in flower development, pollination, and transcription factor activity (Table 4, Additional file 20, Additional file 21). To further explore each of these analytical approaches, we annotated all transcriptomes with gene ontology terms for quantitative comparison. The overall GO representation of each transcriptome qualitatively matches that of other floral transcriptomes [28, 51] and there were no significant deviations in the GO representations between the four species of *Achimenes.* Comparisons of expression patterns for genes involved in anthocyanin and carotenoid biosynthesis, as well as flower development, also allow for further understanding of the temporal and evolutionary patterns of the expressed genes.

### Assembly and consensus transcriptome

Experiments that use transcriptome sequencing have several considerations, including how many replicates to sequence and how much sequencing to perform. The aims of our study were to generate preliminary transcriptome data for four species with three developmental time points in each. Our experiment produced sequenced between 6.3 and 7.2 Gb pairs for each species we sampled (Table 1). From recent transcriptome analyses in other non-model plants, the read generation per sample is commonly 2 to 5 Gb [24, 52–54]. By combining the time point samples in each species, we hoped to provide a large set of reads for *de novo* reference assembly. After combining reads for each time point, the average number of base pairs used for assembly was 6.8 billion (Table 1), similar to these other studies [24, 52–54]. We believe this provides us with an adequate number of reads for initial characterization of our non-model plant subjects. As would be expected, increasing the sequencing depth for a given sample will greatly improve the ability to identify novel and unique transcripts. Future experiments in *Achimenes* will add additional sequencing depth and replicates. We additionally attempted to assemble the best set of transcripts with our data in order to perform comparative analyses relevant to floral developmental processes. Our approach to do numerous assemblies using different parameter settings was an attempt to generate as many complete transcripts as possible. Quality of our assemblies was confirmed by sequence comparison through orthology-based analyses and annotation of transcripts to known genes from model plant species. BLASTx hits to SwissProt proteins that had >80% coverage constituted between 34% and 40% of our assembled transcripts. These factors provide confidence that our experimental approach was able to meet the aims of our study and to provide initial characterization of the floral transcriptomes in non-model plants.

Our study is among the few that use a multiple assembler approach [55–57]. Rather than relying on a single *de novo* assembly program for all contig assembly, we used a combination of Trinity, Velvet, and Oases, to create seven assemblies for each transcriptome that we then merged into a single reference set of contigs. This approach has been used by other studies with success in increases contig length, recovering more unique transcripts, and minimizing sequence redundancy [55–57]. Our approach additionally took advantage of multiple *k*-mer lengths for assembly in Velvet and Oases. Multiple *k*-mer sizes have been demonstrated to assemble more lowly and highly expressed full-length transcripts than using a single *k*-mer size alone [58]. Our Trinity assemblies produced fewer contigs with lower N50 and mean lengths than the Velvet/Oases assemblies (Additional file 1). As the *k*-mer size increases, from 25 to 75, the Velvet and Oases assemblies produced fewer contigs with lower N50 and mean lengths (Additional file 1). Larger *k-*mer sizes also appeared to assemble the largest contigs even though the mean length overall was lower.

Although summaries of the distribution of contig lengths are informative, the goal of transcriptome assembly is not longer sequences, but rather accurate sequences. One metric that remains informative is the proportion of contigs that have significant similarities to known proteins. The difficulty in this measure stems from studies reporting slightly different results using different BLAST parameters and databases. However, nearly 80% of our combined assembly of primary and alternate transcripts had matches in SwissProt or Nr and this value is as high or higher than all other comparable statistics reported in other *de novo* assemblies [20, 24, 28]. Another useful metric is the proportion of the contig and its corresponding best BLAST hit that align to one another. Between 11,420 (27.66%) and 10,281 (35.37%) contigs are covered by at least 75% of their best BLAST hit. These results provide strong evidence that the contigs we assembled in absence of a reference genome largely represent real transcripts and not assembly error.

### Core, shared, and unique genes

Our results indicate that the four *Achimenes* species in our study share a core set of genes expressed during flower development that may also be more broadly shared among other gesneriads. These transcripts code for proteins involved in essential cellular and metabolic functions, such as glycolysis, photosynthesis, and amino acid metabolism (Additional file 12). The transcriptomes also contained “shared” genes, which were observed in two or three of the four target species. There is limited data on how much physiological diversity might be present among such closely related gesneriad species because these taxa have been traditionally defined based on morphological features alone [10, 12]. Therefore, we were interested in what our data may reveal about the relatedness of these closely related taxa. Within the cluster that was unique to all four species (“Shared *Achimenes*”), there was significant overrepresentation of proteins involved in DNA binding and transcription factor activity (Table 3, Additional file 13). This may represent an artifact of our orthogroup clustering approach because our chosen comparison (*Erythanthe*) was a corolla-specific transcriptome rather than whole developing flower as in our samples. We expect that our sampling would capture additional transcripts representing transcription factors involved in calyx, stamen, and ovule development that may be missing from the *Erythranthe* transcriptome. The *Erythranthe* transcriptome is from corolla tissue; therefore, a more complete sampling of the flower would provide a more complete comparison. The overrepresentation of DNA binding activity may also represent an expansion and specialization of transcription factor gene families in *Achimenes* that may have a role in determining many of the unique phenotypes seen. Additional sampling of whole flowers in related species may provide insight into these two possibilities. The remainder of transcripts (approximately one quarter) in each of our four *Achimenes* transcriptomes was found in a single species (Fig. 5). The numbers of transcripts that were putatively species-specific is higher than what we would expect given the close phylogenetic relationships of the four species. Enrichment analyses also did not indicate large numbers of GO terms over- and underrepresented in each species (Additional file 14). Even with the large number of these unassigned transcripts, our assembly pipeline reduced nearly all redundancy by removing identical and closely related sequences.

### Coexpression clustering

Coexpression clustering allows us to identify biological entities (e.g., genes) that share similar profiles across several developmental stages and may help identify groups of genes that are involved in the same biological processes [59, 60]. While we are unable to perform standard analyses of differential expression in the current study (no biological replicates), coexpression clustering provides interesting and useful information on the dynamic temporal changes in gene expression that occur during flower development. Future studies will include additional replicates to perform statistical analyses of differential expression both within and between species of *Achimenes.* Clustering analyses based on metric criteria, such as *k-*means [61] or hierarchical clustering [62], have been broadly used to cluster microarray-based measures of gene expression, as they are rapid, simple, and stable. These approaches require the user to decide on the metric and criterion to be optimized, as well as selecting the appropriate number of clusters, which may not be biologically relevant [63]. We chose an alternative approach, namely probabilistic clustering that uses Poisson mixture models that allowed us a straightforward approach for parameter estimation and model selection for cluster assignment, as well as a per-gene conditional probability of belonging to each cluster. Other model based clustering approaches may also utilize negative binomial (NB) algorithms (such as MBCluster.Seq) [64]. Poisson models have been shown to fit well to data without biological replicates [65] and NB models to data with biological replicates [66]. We therefore use Poisson models to explore patterns of coexpression in our transcriptomes.

Clustering selected between 25 and 34 groups for our transcriptomes that represented genes with shared expression profiles (Fig. 6; Additional file 15; Additional file 17). Enrichment tests validated our approach by identifying significant GO terms that were overrepresented in numerous clusters. A majority of clusters in each species had overrepresented GO terms (Additional file 17, Additional file 18). This clustering approach provides us with groups of genes that are expressed in similar stages that may be linked with particular metabolic or biosynthetic pathways of interest. Coexpression clustering has often been combined in other systems with experimental data or metabolic profiling [67, 68]. Combining clustering data with other approaches has the ability to provide additional support for specific patterns or processes detected from clustering. Obtaining lists of GO terms enriched in coexpression clusters is another useful approach to find patterns within large datasets that can then be used to guide experimental approaches to validate and provide additional support for the patterns seen. Our approach to coexpression clustering differs from commonly used coexpression network approaches that also seek to find biologically interesting clusters of genes sharing similar functional roles. Network analyses, which often use the Weighted Gene Correlation Network Analysis method (WGCNA) [69], usually require at least 15 samples to produce reliable results. Network approaches have been used in other floral transcriptomes to uncover gene networks involved in developing organs [70], floral bud development [71], and pistillate flowering [72]. In future analyses of *Achimenes,* additional replicates and sampling will allow us to perform network-based analyses that may uncover additional gene network modules involved in flower diversification.

### Flower development: spurs

Numerous molecular genetic studies have demonstrated the crucial role of transcription factors in reproductive development of plants. The homologs of many of the genes identified in our study are well known to regulate aspects of flower development in model systems, such as *Arabidopsis*. As expected, we observed an abundance of genes involved in various processes related to flower development, such as the transition to flowering and floral organ identity (Additional file 10). Clear patterns are apparent for genes showing high or low levels of expression during the different developmental time points we sampled. Many studies that have used transcriptome sequencing to understand flower development have focused on sequencing individual floral organs (e.g., petals, stamens, etc.) and comparing them to identify genes differentially expressed between organs [22, 25, 54, 73]. Comparing expression between different tissues has the advantage of being able to identify where individual genes show high or low expression levels. Often these studies focus on a single species. Our aims for the current study were instead to investigate and compare the floral transcriptome in many closely related species that exhibit very diverse flowers. The advantage of our approach is the ability to begin understanding how gene expression differences may contribute to phenotypic differences among closely related species. We identified over 100 transcripts likely involved in flower developmental processes (Additional file 10). These transcripts in *Achimenes* largely show similar expression patterns seen in other flowering plants [22, 25, 74]. The orthologs of many well-known MADS-box genes (e.g., *AP1, AP3, PI,* and *AG*) are crucial for orchestrating floral organ identify [75, 76]. The expression patterns of these genes follow what we might expect given when the different floral organs are developing in *Achimenes* flowers (Additional file 10). For instance, the A-class genes *AP1* shows high expression during the bud stage when sepals are developing and the B-class genes *AP3* and *PI* have increased expression during D stage when petals are developing (Additional file 10). Elaboration of the petals to produce different shapes and widths likely involves genes outside these MADS-box genes [77].

Some species of *Achimenes* (including *A. patens*) exhibit a unique spur-like outgrowth of the petal tube that extends in opposition to the tube opening (Fig. 1). This petal spur has evolved independently at least three times in *Achimenes*, mostly in butterfly-pollinated species where the flower is presented at a downward angle (Fig. 1). The purpose of this petal spur in *Achimenes* has yet to be elucidated; it differs from the spurs in other lineages (such as columbines, *Aquilegia*) by not containing nectary tissue [10]. The genetic factors influencing the development of spurs have not yet been fully understood. Recent transcriptome sequencing of developing spur tissue in *Aquilegia* identified several candidate genes for this process, including homologs of *TCP4, GRF1,* and many other genes that contribute to cell proliferation and auxin signaling [37]. We see an increased level of gene expression for *TCP4* in *A. patens* in the stages where spur growth is seen while this gene in the other three species remains much lower (Fig. 4). We also see an increase in gene expression of *STY1* and *ARF8* in *A. patens*, similar to what was reported in *Aquilegia* (Fig. 4). With the patterns seen in *A. patens* relative to the other species, we can hypothesize that *TCP4* may be playing a significant role in the development of the petal spur. KNOX genes, particularly *STM*, have also been hypothesized to be important players in petal spur development in *Antirrhinum* and *Linaria* [78, 79]. Overexpression of KNOX genes in *Antirrhinum* produced spur-like outgrowths in the floral tube [78], while KNOX genes in *Linaria* displayed increased expression in petal spur tissue [79]. Our expression estimates for *STM* across *Achimenes* do not offer as clear a pattern as *TCP4*; *STM* gene expression patterns are similar across several species (Fig. 4). The pattern of *STM* expression is similar in both *A. patens* and *A. misera* (Fig. 4). Testing the functional roles of *TCP4* and *STM* will be important in future work to determining which is more likely to be important for petal spur growth in *Achimenes*.

### Flower color: anthocyanins

Differences in flower color are one of the most distinguishing characters that separate *Achimenes* species. Flowers across the genus display an amazing array of colors and color patterns, including species with white, yellow, red, blue, and purple pigmentation [10, 12] (Fig. 1). The primary pigment in flowers of *Achimenes* and most angiosperms are anthocyanins, a class of flavonoids that represent a large group of secondary metabolites [80]. The types of pigments present in floral tissue vary across *Achimenes* species, with all taxa containing anthocyanins and several containing a mix of anthocyanins and carotenoids. Anthocyanins contribute hues of blues, purples, and reds due primarily to production of pelargonidins, cyanidins, and delphinidins [80]. In plants, the biochemistry of the ABP is very well studied and understood in both model systems (e.g., *Arabidopsis*) [81] and non-model systems (e.g., *Aquilegia, Mimulus,* and *Iochroma*) [82–86]. While the biochemical reactions involved in the ABP are well understood, further research aims at understanding how the genetics of the pathway contributes to species differences in pigment production and the role it plays in adaptive evolution. The ABP is composed of 7 structural loci, with many of the earliest steps highly conserved in plants due to their role in producing precursor products involved in defense and UV protection [80, 81] (Fig. 2). The downstream pathway splits into 3 branches that lead to production of red pelargonidins, purple cyanidins, and blue delphinidins [80]. Flux down any of these branches is largely determined by the activity of two enzymes: *F3′H* and *F3′5′H*. Downregulation or inactivation of these enzymes can cause flux to be redirected down a different branch, resulting in a different flower color.

Several possible routes to produce variation in anthocyanin production exist, including gene loss or transcriptional regulation. One predominant example seen numerous times across flowering plants is the shift from blue-colored flowers to red-colored flowers that is closely associated with a shift from bee pollination to bird pollination [84, 85, 87–90]. These studies have implicated the downstream enzymes of the ABP (particularly *ANS*, *DFR*, *F3′H*, and *F3′5′H*) being involved in flower color transitions. Primarily, two often predictable routes have been suggested for the transition from blue to red anthocyanin pigment production: acquisition of mutations in *DFR* that alter its substrate specificity [84–86] or altered expression of *F3′H* and *F3′5′H* resulting from *cis-* of *trans*-regulatory mutations [84, 88–90]. Given the constrained structure of the ABP and the few demonstrated genetic changes involved in flower color transitions, our focus in *Achimenes* lays in genetic changes involving the enzymes *DFR*, *F3′H*, and *F3′5′H*, as well as the R2R3-Myb transcription factors that regulate the ABP [86, 91].

In *Achimenes*, multiple transitions from blue to red exist [10], and there also exists at least one likely red-to-blue flower color transition on the branch leading to *A. cettoana* (Fig. 1). This type of transition is exceedingly rare in plants and has few documented explanations. The transition of blue-to-red is more common and often involves predictable changes to key enzymes of the ABP, including *DFR*, *F3′H*, and *F3′5′H* (see Discussion above). One such case of red-to-blue flower color transition involves a gene duplication of *F3′H* and neofunctionalization to regain the role of *F3′5′H* in Asteraceae [92, 93]. A similar gene duplication event is not found when the gene trees are examined for *F3′H* and *F3′5′H* (Additional file 7), suggesting that changes in gene expression are more likely involved in a red-to-blue color transition in *Achimenes*.

We captured transcripts of core downstream enzymes of the ABP from all 4 transcriptomes, each with appreciable expression levels that show an increase from B to A stage (Fig. 2). Several patterns of expression emerge from the data. Both *A. cettoana* and *A. patens* have increased expression of *F3′5′H,* the enzyme responsible for directing the flux of the pathway toward delphinidin production (Fig. 2). These flowers are blue and purple, so this pattern is what we might expect to see. Expression levels of the enzymes in *A. misera* are much lower, which we might also expect given that this flower produces very little pigment except in areas of the corolla throat (Fig. 2). Expression of *F3′5′H* is much lower in *A. erecta*, the red-flowered species (Table 3) and this pattern follows the pattern seen in other systems [83, 85]. The possible explanation for how the red-to-blue color transition could have occurred in *Achimenes* will require more detailed studies than those presented here, but given that we see expression of all ABP enzymes, it is possible that differences in anthocyanin production are due to genetic changes in the transcription factors that regulate the pathway, not in loss of function mutations as found in other systems [83–85, 94]. Additionally, *Achimenes* species tend to produce anthocyanins in both floral and vegetative tissue [12]. This coupled with the captured expression of the ABP enzymes may suggest that flower color transitions may involve a change to substrate specificity in *DFR* or in the downregulation of *F3′H* and *F3′5′H* enzymes in red flowers through *trans-*activating mutations.

It is interesting to find that several of the ABP enzymes are coexpressed together and in three species (*A. cettoana*, *A. misera*, and *A. patens*) they are coexpressed with candidate R2R3-Mybs that we identified (Additional file 19). In *A. cettoana*, the candidate R2R3-Myb is coexpressed with *F3′5′H*, the enzyme that directs the metabolic flux of the pathway toward the production of delphinidins (Fig. 2). Another candidate R2R3-Myb in *A. patens* was coexpressed with *F3H*, *F3′5′H*, and *ANS* (Additional file 19). With this pattern in these two species, we might hypothesize that the candidate R2R3-Mybs are involved in transcriptional regulation of the ABP to produce delphinidin pigments. This is what we would expect given the blue and purple flower color in these species. In *A. misera*, one candidate R2R3-Myb was coexpressed with *ANS* and might be involved in regulating more downstream parts of the ABP (Additional file 19).

The role of R2R3-Myb transcription factors in regulating various steps of the ABP has been well studied in numerous plants [86, 91, 95] and the possible role of these transcription factors in *Achimenes* will need to be studied further. We identified putative proteins in *Achimenes* with high-similarity to R2R3-Mybs that have experimental evidence indicating their role in regulating anthocyanin accumulation (Additional file 11). These *Achimenes* R2R3-Mybs are closely related to homologs recently identified in *Erythranthe* [86] as well as homologs from *Petunia* [96] and *Antirrhinum* [97]. We can hypothesize that these R2R3-Mybs from *Achimenes* may function similarly to regulate expression of the ABP given their close similarity to other homologs as well as their coexpression patterns.

### Flower color: carotenoids

Carotenoids are important pigments that carry out functions in protecting the photosynthetic apparatus from photooxidative damage and acting as accessory pigments in light harvesting [98]. In non-photosynthetic tissues, carotenoids are usually synthesized as secondary metabolites and accumulate in chromoplasts, providing the yellow, orange, and red colors in many flowers, thus serving an important function in the ecology and evolution of plants by attracting pollinators and seed dispersers [99]. In many *Achimenes* species, carotenoids are found throughout the corolla; while in other species carotenoid production is limited to the corolla throat (as in *A. erecta* and *A. misera*). Few species, including *A. cettoana* and *A. patens,* do not appear to produce carotenoids in the corolla tissue and only produce anthocyanins.

We identified putative enzymes in the plant carotenoid biosynthetic pathway (CBP) in each of our transcriptomes (Fig. 3). The CBP splits into two branches: the α-carotene branch (Fig. 3) and the β-carotene branch (Fig. 3). Biochemical studies of floral carotenoids are lacking in Gesneriaceae, therefore we cannot confidently assess which carotenoids are present in *Achimenes* corollas without doing biochemical experiments. Our expression estimates of the CBP enzymes indicate activity of all the core enzymes in each species (Fig. 3). Some species of *Achimenes,* including *A. cettoana* and *A. patens,* contain no carotenoids in the corolla and lower expression of the CBP enzymes in these species may reflect carotenoid accumulation in sepals and pollen. In other systems, particularly *Erythranthe*, all floral carotenoids are on the β-carotene branch [100]. We find lower levels of 2 enzymes exclusive to the α-carotene branch (*LCYE* and *CYP97*) compared to the other enzymes found on the β-carotene branch (*LCYB*, *BCH*, *ZEP*, *NXS*, and *NCED*; Fig. 3). These results may indicate that *Achimenes* and other gesneriad species are primarily producing floral carotenoids via the β-carotene branch, but further biochemical characterization and experimental studies will need to be undertaken to support this conclusion.

In general, expression estimates of CBP enzymes are lower in *A. cettoana* and *A. patens* (Fig. 3) and both of these butterfly-pollinated species contain little to no visible carotenoid pigment accumulation in their corolla. Flavonoids (like anthocyanins) absorb UV light and carotenoids reflect UV light. Presence of anthocyanins in the petal lobes and absence in the petal tube may reflect the common use of a ‘bulls-eye’ UV pattern to attract insect pollinators. In contrast, *A. erecta* and *A. misera* contain visible amounts of carotenoids in the corolla tube. Bee-pollinated *A. misera* flowers have a clear nectar guide on the ventral petal formed by the accumulation of carotenoids, an important trait for successful bee pollination [101, 102]. Bird-pollinated flowers, like *A. erecta*, often contain combinations of anthocyanins and carotenoids, with red anthocyanins preventing visitation by bees [103]. Taken together, the pigments contributing to flower color in *Achimenes* are important for determining what pollinators visit. Despite butterfly- and bee-pollinated flowers likely containing a nectar guide, in *A. cettoana* and *A. patens* it appears to be due to flavonoids, while in *A. misera* it appears from both flavonoids and carotenoids.

The regulation of carotenoid pigmentation in flowers is less well understood than the regulation of the ABP. An R2R3-Myb transcription factor, Reduced Carotenoid Pigmentation 1 (*RCP1*), has been the only transcription factor identified to be involved in flower-specific carotenoid biosynthesis [95]. Our analyses identified 9 transcripts with similarity to *RCP1* (Additional file 11). However, when we look at patterns of coexpression we only find one candidate (in *A. erecta*) being coexpressed with any of the enzymes of the CBP (Additional file 19). Future genetic experiments will be important to elucidating the transcriptional regulation of this network in *Achimenes* flowers. So far, we have identified potential candidate transcription factors, but their specific function will need to be further explored.

### Adaptive evolution

The evolution of floral form among the four *Achimenes* species is likely influenced by differences in pollinator availability and preferences. Within the group, there are distinct floral forms that correspond closely with different pollination syndromes [10]. Highly dimensional quantitative data of floral morphology and qualitative data of color and petal spur size can be reduced into groups that correspond closely to different pollinators. Flowers of *Achimenes* are visited by a number of insects (bees, Apidae; euglossine bees, Euglossini; butterflies, Lepidoptera) and hummingbirds (Trochilidae) [13]. Observations of pollinator visitation to four *Achimenes* species provide evidence for the use of pollination syndromes to separate floral form into unique groups [13]. Linking protein evolution to the convergent evolution of these different pollination syndromes may provide evidence for shared or different genetic routes to these forms. Previous studies have suggested the pathways involved in pigment production, particularly anthocyanins, are involved in pollination syndrome transitions [45, 74, 75].

Our selection analyses found numerous genes showing significant signs of molecular evolution (Table 4, Additional files 18, Additional file 21). However, our analyses did not provide statistical over- or underrepresentation of any GO terms within the set of proteins with sites under positive selection. We do find a number of proteins involved in various processes during flower development that might be involved with floral diversification (Table 4). Many genes have GO terms associated with them involving the regulation of flower development, anatomical structure development, and transcription factor activity, among others (Additional files 18, Additional file 21).

None of the core enzymes of the ABP or the CBP that we identified were under positive selection. However, a protein annotated as *DFR* was identified from *A. erecta* (Table 4). The sequence of this protein shares similar motifs with the *DFR* enzyme we identified above, but is not the same transcript (Additional file 6). Given its annotation and similarities it is likely involved in anthocyanin production, but possibly in a different step of the ABP than the core part of the pathway we considered here. Another protein was identified in *A. erecta* and annotated as *ABCB19* (ABC transporter B family member 19; Table 4), an auxin efflux transporter with roles in mediating anthocyanin accumulation in floral tissue [104]. Additionally, in *A. cettoana*, a protein annotated as *5MAT1* (malonyl-coenzyme:anthocyanin 5-O-glucoside-6”’-O-malonyltransferase; Table 4) was also identified with a role in catalyzing the transfer of a malonyl group to the pelargonidin pigment classes [105]. Like *DFR*, both *ABCB19* and *5MAT1* are likely involved in anthocyanin biosynthesis, albeit outside of the core pathway. Other studies have found signatures of positive selection in the core ABP enzymes [106], but in the current study we do not detect any significant evidence.

Some interesting genes involved in flower development were additionally identified to be under positive selection. In *A. patens, HDA19* (histone deacetylase 19) is a protein involved in epigenetic repression and plays an important role in transcriptional regulation, particularly the repression of several A- and E-class MADS-box genes that control sepal and petal identity [107]. The role of this histone deacetylase in the epigenetic modification of floral developmental programs in *A. patens* is not immediately apparent; therefore, additional studies will be useful to understand the potential myriad roles this gene may play in development. Another protein under selection identified from *A. misera*, a homolog of *FRI* (frigida), is involved in flowering time transition [108]. Allelic variation in *FRI* was demonstrated in *Arabidopsis* to be important for natural variation in flowering time across different latitudes [108]. Flowers are produced on *A. misera* nearly constantly during the growing season and the potential role of *FRI* in development will need to be assessed in further experiments.

With expanded sampling of additional *Achimenes* species, our analyses of positive selection will be more robust than those presented here. We were able to include sequences from five species (4 ingroup and 1 outgroup) and compare gene families that contained members from each of those species. Our use of site-models allows our detection of specific amino acids within the protein that may be undergoing positive selection [48, 49]. Evolutionary change can also happen in the regulatory region of genes, which may affect the level, timing, and location of gene expression. Without a genome reference to look for upstream and downstream mutations that may affect particular genes, we are unable to currently look at these regions for their effect on genes involved in floral diversification.

## Conclusions

The newly sequenced, assembled, and annotated floral transcriptomes for *Achimenes. cettoana, A. erecta, A. misera,* and *A. patens* provide valuable genomic resources to study the molecular mechanisms of development, adaptation, and speciation between closely related species. Comparative analyses of closely related taxa are important for understanding the molecular mechanisms involved in the evolution and diversification of lineages. The diversity of floral forms in *Achimenes* is hypothesized to correspond to pollinator-driven preferences toward different shapes, colors, and orientations to provide successful pollination and fertilization [7, 10]. Large similarities between the floral transcriptomes in closely related species with diverse floral phenotypes suggests that these visible differences are, in part, due to changes in a small set of genes. Combining analyses of sequence orthology, gene expression, and molecular evolution have provided initial candidates for future analyses into the diversification of floral form. Exploration of the expression patterns for genes relating to flower color and flower shape has provided interesting patterns corresponding to the floral form of each species. Patterns of expression for genes involved in anthocyanin and carotenoid biosynthesis indicate that flower color transitions may be due to changes in a small set of genes, some of which are coexpressed together. The datasets presented here also contribute to the growing number of available genomic resources for species in the family Gesneriaceae [50, 109–112] that are study organisms for desiccation tolerance, flower development, and leaf development. Together, these newly developed genomic tools provide a valuable resource for ecological and evolutionary genomics projects, serving as a starting point to begin understanding phenotypic variation and the evolutionary genetic forces driving variation across species and populations in the Gesneriaceae and other tropical plant lineages.

## Methods

### Plant material

Flower shape in *Achimenes* can take many forms, including funnelform, salverform, tubular, and a number of intermediate forms (Fig. 1). Primary flower color is also quite variable and is represented by flowers of white, purple, pink, red, blue, and yellow colors (Fig. 1). We chose to sample species broadly across *Achimenes* for the present study in order to develop initial resources for understanding the genomic basis for flower diversification. Our sampling includes *A. cettoana*, a butterfly pollinated species with purple-blue salverform flowers (Fig. 1), *A. erecta*, a hummingbird pollinated species with red salverform flowers (Fig. 1), *A. misera*, a bee pollinated species with small, white funnelform flowers with a purple throat (Fig. 1), and *A. patens*, a butterfly pollinated species with large, purple-pink salverform flowers and a noticeable petal spur (Fig. 1). These four species represent most of the common flower shapes and colors seen in the genus, and while they do not represent all of the possible floral forms, they present us with a starting point to guide future studies. Vouchers of each sampled species are deposited in the WR herbarium with the following identification numbers: *A. cettoana*, WR0155; *A. erecta*, WR0156; *A. misera*, WR0157; *A. patens*, WR0158.

Three stages of flower development were sampled so that temporal changes in gene expression could be studied. ‘Immature Bud’ (B) stage was the smallest flower buds that could be distinguished from vegetative buds (Fig. 1). ‘Stage D’ (D) were larger flower buds that were beginning to accumulate pigmentation, the cells in the corolla tube are elongating, and the petal spur (as in *A. patens*) is beginning to develop (Fig. 1). ‘Pre-Anthesis’ (A) flower buds were the largest and fully pigmented and were collected one-day before anthesis (Fig. 1). Given that the different species have different flowering times, these stages are determined from qualitative observations. Plants were grown in greenhouse conditions under natural daylight, controlled temperature ranging from 27 to 32 °C, and >80% humidity. For all experiments, plant material was harvested directly into liquid nitrogen and subsequently stored at -80 °C. To obtain enough fresh material for RNA extraction, between 2 and 5 flower buds were sampled from an individual plant.

### Library preparation and sequencing

Total RNA was isolated from developing flower buds of *Achimenes* by grinding 50–100 mg of tissue frozen in liquid nitrogen. RNA was then extracted using the Qiagen RNeasy Plant Mini Kit (Qiagen, Valencia, CA) following the manufacturers instructions. To avoid genomic DNA contamination, RNA was treated with Rnase-free Dnase I (Thermo Fisher Scientific, Waltham, MA). The RNA integrity was assessed by visualization in 1.0% agarose gels and RNA Integrity Number (RIN) as measured by an Agilent 2100 BioAnalyzer (Agilent, Santa Clara, CA). Ribosomal-depleted RNA samples were prepared using the Ribo-Zero rRNA Removal Kit for plant leaf material (Illumina, San Diego, CA). Sequencing libraries were constructed using the TruSeq RNA-seq sample prep kit from Illumina (Illumina, San Diego, CA) according to manufacturers instructions. All stages of library preparation were performed at the Genome Sequencing and Analysis Facility (GSAF) at the University of Texas (Austin, TX). RNAseq libraries were quantified using a BioAnalyzer 2100 High Sensitivity DNA chip and pooled based on nM concentrations. Individual libraries were uniquely barcoded, multiplexed, and sequenced for 100 bp paired-end reads (2 x 100 bp) using one lane on the Illumina HiSeq2500 at the GSAF.

### *De novo* assembly

Raw 100 bp paired-end Illumina reads were sorted by barcode and assessed for quality using the tools implemented in FastQC [113]. The 3′-ends of the reads were quality trimmed using FASTX-Toolkit [114], removing any reads that contained bases with Phred scores less than 20. We also discarded any low quality reads less than 50 bp long or with less than 80% of bases having a Phred score greater than 20. Contaminating Illumina adapter sequences and primers were also trimmed.

Three *de novo* assemblers were used to construct a robust set of contigs using different algorithms and *k*-mer sizes: Trinity (Tr), Velvet (Vt), and Oases (Oa) [115–117]. Data from the three developmental stages in each species were concatenated prior to *de novo* reference assembly. To provide sets of assembled transcripts, we employed multiple assemblers using a range of *k*-mer sizes. For Tr assembly, we used forward-reverse read orientation (--SS_lib_type FR) with the default *k-*mer size of 25. For Vt assembly, we utilized a multiple *k*-mer approach, with separate assemblies performed for *k-*mer sizes 25, 35, 45, 55, 65, and 75, and specifying a library insert size of 150 (-ins_length 150). Each Vt *k*-mer assembly was further assembled using Oa under the default settings.

In order to reduce the redundancy of assemblies and create sets of primary and secondary transcripts, all assemblies were subjected to the EvidentialGene tr2aacds pipeline [118]. Merged assemblies were produced using the seven *de novo* assemblies generated previously. Each *de novo* assembly for each species was generated using the three tissue samples from the same species. The EvidentialGene pipeline selects a ‘best’ set of *de novo* assembled transcripts, based on coding potential, from a pool of such sequences. The algorithm first infers the coding DNA sequences (CDS) and amino acid sequences for each sequence, and then removes redundant sequences using the amino acid information by choosing the best coding sequences from amongst identical sequences with fastanrdb (exonerate-2.2.0) [119] and CD-HIT-EST [120]. Self-on-self BLASTn is then implemented to identify highly similar sequences. The alignment data and CDS/protein identities are then used to select and output transcripts classified as ‘main’ (primary; the best transcripts with unique CDS) or ‘alternate’ (possible isoforms), and another set classified as ‘dropped’ which did not pass the internal filters of the pipeline. The chosen primary and alternate contigs were used for further analyses and annotation.

### Functional annotation

To annotate transcripts, we conducted a BLAST search of all unique ‘primary’ transcripts against the SwissProt database (BLASTx, E-value = 1e-06) [30], NCBI non-redundant (Nr) protein database (BLASTx, E-value = 1e-06) [31], and Plant Non-coding RNA Database (BLASTn, E-value = 1e-06) [32]. Additionally, the ‘alternate’ transcripts sets were searched against the SwissProt database for annotation. For each sequence we retained the top five BLAST hits for subsequent analysis. We placed first priority to the SwissProt database hits for annotation, followed by the Nr and PNRD databases because the SwissProt database contains more GO identities associated with the protein hits than either the Nr or PNRD databases. Sequences with a match in either the SwissProt or Nr database were subsequently annotated with GO terms [33] as implemented in Blast2GO v.3.0 [121]. InterProScan was used to scan transcripts for domain and motif information that may provide additional GO identities not attributed using blastx hits alone [33]. GO terms were assigned based on BLAST hits and InterProScan results to cover three types of terms: BP, CC, and MF. We additionally integrated the Second Layer Concept of Myhre et al. [35] (ANNEX augmentation) to identify, given the molecular function, biological processes where the molecular functions are involved, and cellular components where they are active. Finally, GO terms were simplified to a smaller set of high-level GO terms (GO slims) [122]. We obtained GO slims through Blast2GO with the plant slims developed by the *Arabidopsis* Information Resource [122]. Additionally, non-coding ribosomal RNAs and transfer RNAs were detected using RNAmmer [123] and tRNAscan [124], respectively. We tested for significant differences in sequence representation for GO categories between all species with a Chi-square test followed by using False Discovery Rate (FDR, *α* = 0.05) adjusted *p*-values [125].

Specific enzymes related to anthocyanin pigment production were identified through hidden markov models (HMM) built and trained in HMMER [36]. We searched our assemblies for proteins identified as homologs to *ANS*, *DFR*, *F3′H*, and *F3′5′H*. Protein sequences from other studies were downloaded from GenBank (Additional file 4), aligned using MUSCLE [126], and used to create HMM profiles. These HMM profiles were then used to search our reference transcriptome to identify possible candidates proteins. These candidate proteins were then aligned with candidates from other studies (Additional file 4) using MUSCLE and visually inspected to identify and correct misaligned regions. Finally, these alignments were used to construct neighbor-joining trees in Geneious version R9 [127] with branch support assessed by performing 100 bootstrap replicates.

Putative proteins involved in flower development, carotenoid biosynthesis, and petal spur development (taken from [37]) were identified by BLASTp searches against *Arabidopsis* homologs downloaded from the UniProt database (Additional file 4; www.uniprot.org). The criteria used to determine the best-hit transcript were (in order): bit score, E-value, and percent identity.

Members of the R2R3-Myb transcription factor family that may be involved in floral pigmentation were identified using HMM models built and trained in HMMER [36]. Proteins with experimental evidence supporting their role in the transcriptional regulation of floral pigmentation were downloaded from GenBank (Additional file 4; https://www.ncbi.nlm.nih.gov/genbank/). The proteins were first aligned using MUSCLE [126], and then the conserved Myb domains were extracted, re-aligned using ClustalW [128], and used to construct a neighbor-joining tree in Geneious version R9 [127] with branch support assessed by performing 100 bootstrap replicates.

### Orthogroup identification

We next identified conserved orthogroups from the sets of translated proteins identified in each *Achimenes* species using OrthoFinder v.0.3.0 [129]. This method solves the problem of gene length bias in BLAST searches by normalizing the bit scores by both gene length and phylogenetic distance and outperforms the more commonly used OrthoMCL in accuracy and speed [129]. Orthologs and paralogs were determined for each species individually as well as in five-way comparisons. In the comparative analyses, we used a corolla transcriptome from *Erythranthe lewisii* LF10 (15 mm corolla; available from http://www.monkeyflower.uconn.edu/resources) as comparison [86]. We chose *E. lewisii* for comparison because it is a flower-specific transcriptome that is phylogenetically close to *Achimenes* (both are members of the Order Lamiales). Protein coding sequences were produced for *E. lewisii* using TransDecoder v.2.0 [130], under default settings.

### Quantifying and comparing gene expression patterns

Trimmed, high-quality reads from individual stage-specific samples (B, D, and A) were independently mapped onto each primary reference transcriptome using the ungapped alignment software bowtie [42]. We used the abundance of reads derived from each locus to estimate gene expression and calculate transcripts per kilobase million (TPM) values with the program RSEM (RNA-Seq by Expectation Maximization) [43]. The numbers of reads mapped per library were normalized by the trimmed mean of M-values normalization method (TMM) [131]. Genes were considered expressed in a developmental stage if they had a normalized TPM ≥0.01 in that stage. Expression estimates for floral developmental genes in individual species were transformed to Z-scores for heatmap representations.

Transcripts with estimated expression values ≤0.01 were removed prior to clustering. To cluster sets of co-expressed genes within each species, we performed clustering using HTSCluster [44]. Unlike other commonly used clustering algorithms (e.g., k-means, hierarchical), HTSCluster is a model based clustering approach that uses Poisson mixture models to cluster sequences using expression estimates and selects the appropriate number of clusters using slope heuristics (Djump and DDSE) [46]. We ran HTSCluster using the EM [45] algorithm for parameter estimation and tested cluster numbers ranging from *K* = 1, 2, …, 60. From 5 independent runs, we selected the model and associated cluster number that had the highest log-likelihood. We used both the Djump and DDSE criteria to select the number of clusters for each run. The degree of certitude in cluster assignment was additionally evaluated using the maximum conditional probabilities of cluster membership for the genes assigned to each cluster.

### Detecting genes under selection

Each orthogroup identified with the OrthoFinder five-way analysis was run through a pipeline to identify protein sites potentially undergoing selection. The pipeline first takes the CDS sequences and inferred homology relationships and filtered these based on numeric, phylogenetic, and quality criteria to remove spurious data. We chose to keep proteins having a complete coding region (strings in multiples of 3), a minimum of 5 species and 5 sequences, and mean sequence divergence of ≤60%. Each satisfactory orthogroup then undergoes multiple sequence alignment using MUSCLE [126], protein-guided codon alignment using TrimAl [132], and phylogenetic tree reconstruction using dnaml from Phylip [133]. Finally these orthogroups are analyzed for signatures of selection using the site-models implemented in PAML v.4.6 [134]. For our analyses, we used the M1a (neutral), M2a (selection), M7 (beta), and M8 (beta + ω) models implemented in codeml [48, 49]. Model M1a was compared to M2a and M7 was compared to M8. Significance differences in model fit for each comparison were assessed using a likelihood ratio test followed by FDR correction for multiple hypothesis testing (*α* = 0.05).

### Gene ontology enrichment analyses

We used the FatiGO [135] package as integrated with Blast2GO to assess enrichment of GO terms in the proteins identified during 1) orthogroup clustering, 2) coexpression clustering, or 3) detection of sites under positive selection. Previously for each *Achimenes* transcriptome, we obtained a list of annotated transcripts with associated GO identities. This information was then divided into three GO maps based on the three GO domains: 1) BP, 2) CC, and 3) MF. Each analysis was performed using a two-tailed Fisher’s Exact Test using FDR-corrected *p*-values (*α* ≤ 0.05). Both over- and underrepresented GO terms were identified for each cluster or group relative to the whole transcriptome background.

## Additional files

Additional file 1: Table S1. Detailed sequencing and assembly statistics. **A.** Trinity assembly. **B.** Velvet and Oases assemblies. **C.** EvidentialGene merged assembly. (XLSX 41 kb)
Additional file 2: Table S2. Number of contigs detected as rRNAs and tRNAs in each transcriptome. (XLSX 33 kb)
Additional file 3: Figure S1. Counts and proportion of level 2 Gene Ontology annotations for *Achimenes* transcriptomes. (PDF 361 kb)
Additional file 4: Table S3. Protein homologs downloaded from GenBank used for HMM profile searches. **A.** *ANS.* **B.** *DFR*. **C.** *F3′H* and *F3′5′H*. **D.** R2R3-Mybs. (XLSX 48 kb)
Additional file 5: Figure S2. Neighbor-joining tree of anthocyanidin synthase (*ANS*) gene family. Putative *Achimenes ANS* orthologs are highlighted in red. Bootstrap support >50 are indicated above branches. (PDF 455 kb)
Additional file 6: Figure S3. Neighbor-joining tree of dihydroflavonol 4-reductase (*DFR*) gene family. Putative *Achimenes DFR* orthologs are highlighted in red. Boostrap support >50 are indicated above branches. (PDF 330 kb)
Additional file 7: Figure S4. Neighbor-joining tree of flavonoid 3′-hydroxylase (*F3′H*) and flavonoid 3′,5′-hydroxylase (*F3′5′H*) gene family. Putative *Achimenes F3′H* and *F3′5′H* orthologs are highlighted in red and blue, respectively. Bootstrap support >50 are indicated above branches. (PDF 1175 kb)
Additional file 8: Figure S5. xAligned protein sequences for *Achimenes F3′H, F3′5′H,* and *DFR.* Amino acid substitutions between the sequences of *A. cettoana, A. erecta, A. misera,* and *A. patens* are highlighted in blue, red, black, and pink, respectively. A, *F3′H*; B, *F3′5′H*; C, *DFR. (PDF 3121 kb)*

Additional file 9: Table S4. Homologs of flower development genes identified in *Achimenes*. Included are the expression domain, primary role, and gene family. (XLSX 60 kb)
Additional file 10: Figure S6. Expression of genes involved in flower development in *Achimenes*. (PDF 346 kb)
Additional file 11: Figure S7. Neighbor-joining tree of R2R3-Mybs in *Achimenes*. Putative orthologs involved in anthocyanin and carotenoid biosynthesis are highlighted in blue and orange, respectively. Bootstrap support >50 are indicated above branches. (PDF 921 kb)
Additional file 12: Table S5. Significantly enriched Gene Ontology terms for sequences in the “Core” transcriptome after orthogroup classification. Terms are enriched if they have FDR-corrected *p*-values < 0.05 (Fisher’s Exact Test). Those terms that are overrepresented when all species are analyzed together (“Combined” column) are in bold. (XLSX 16 kb)
Additional file 13: Table S6. Significantly enriched Gene Ontology terms for sequences in the “Shared *Achimenes*” clusters after orthogroup classification. Terms are enriched if they have FDR-corrected *p*-values < 0.05 (Fisher’s Exact Test). Those terms that are overrepresented when all species are analyzed together (“Combined” column) are in bold. (XLSX 48 kb)
Additional file 14: Table S7. Significantly enriched Gene Ontology terms for sequences that were unassigned during orthogroup classification. Terms are enriched if they have FDR-corrected *p*-values < 0.05 (Fisher’s Exact Test). (XLSX 38 kb)
Additional file 15: Figure S8. Coexpression clusters for *Achimenes* determined using Poisson mixture models. Gene profiles are depicted as boxplots. Conditions are as follows: 1, Bud stage; 2, Stage D; and 3, Pre-Anthesis stage. A, *A. cettoana*; B, *A. erecta*; C, *A. misera*; D, *A. patens*. (PDF 4192 kb)
Additional file 16: Figure S9. Maximum conditional probability of cluster membership assigned by coexpression clustering using Poisson mixture models. A, *Achimenes cettoana*; B, *A. erecta*; C, *A. misera*; D, *A. patens*. (PDF 1176 kb)
Additional file 17: Table S8. Detailed model selection statistics and Gene Ontology enrichment for coexpression clustering. **A.** *A. cettoana*. **B.** *A. erecta*. **C.** *A. misera*. **D.** *A. patens.* (XLSX 54 kb)
Additional file 18: Table S9. Gene Ontology enrichment for coexpression clustering. **A.** *A. cettoana.* **B.** *A. erecta.* **C.** *A. misera.* **D.** *A. patens. (XLSX 228 kb)*

Additional file 19: Table S10. Coexpression clusters for candidates involved in anthocyanin biosynthesis, carotenoid biosynthesis, and spur development. (XLSX 40 kb)
Additional file 20: Table S11. Annotated proteins with sites under selection using PAML for M1a vs. M2a comparison. **A.** *Achimenes cettoana*. **B.** *Achimenes erecta.* **C.** *Achimenes misera.* **D.** *Achimenes patens.* **E.** *Erythranthe lewisii*. (XLSX 60 kb)
Additional file 21: Table S12. Annotated proteins with sites under selection using PAML for M7 vs. M8 comparison. **A.** *Achimenes cettoana.* **B.** *Achimenes erecta.* **C.** *Achimenes misera.* **D.** *Achimenes patens.* **E.** *Erythranthe lewisii*. (XLSX 75 kb)

## Abbreviations

ABP: Anthocyanin biosynthetic pathway
*ANS*: Anthocyanidin synthase
BP: Biological process
CBP: Carotenoid biosynthetic pathway
CC: Cellular component
CDS: Coding DNA sequences
DDSE: Data driven slope estimation
*DFR*: Dihydroflavonol 4-reductase
Djump: Dimension jump
EM: Expectation maximization algorithm
*F3′5′H*: Flavonoid 3′,5′-hydroxylase
*F3′H*: Flavonoid 3′-hydroxylase
FDR: False discovery rate
GO: Gene ontology
HMM: Hidden markov models
MF: Molecular function
NB: Negative binomial
Nr: NCBI non-redundant protein database
Oa: Oases assembly
PNRD: Plant Non-coding RNA Database
TMM: Trimmed mean of M-values normalization method
TPM: Transcripts per kilobase million
Tr: Trinity assembly
Ve: Velvet assembly
