# Supplementary figures and images for: Comparative transcriptome analyses of flower development in four species of Achimenes (Gesneriaceae)
Source: BMC Genomics. 2017 Mar 20;18:240. doi: 10.1186/s12864-017-3623-8 (PMC5359931; doi:10.1186/s12864-017-3623-8)

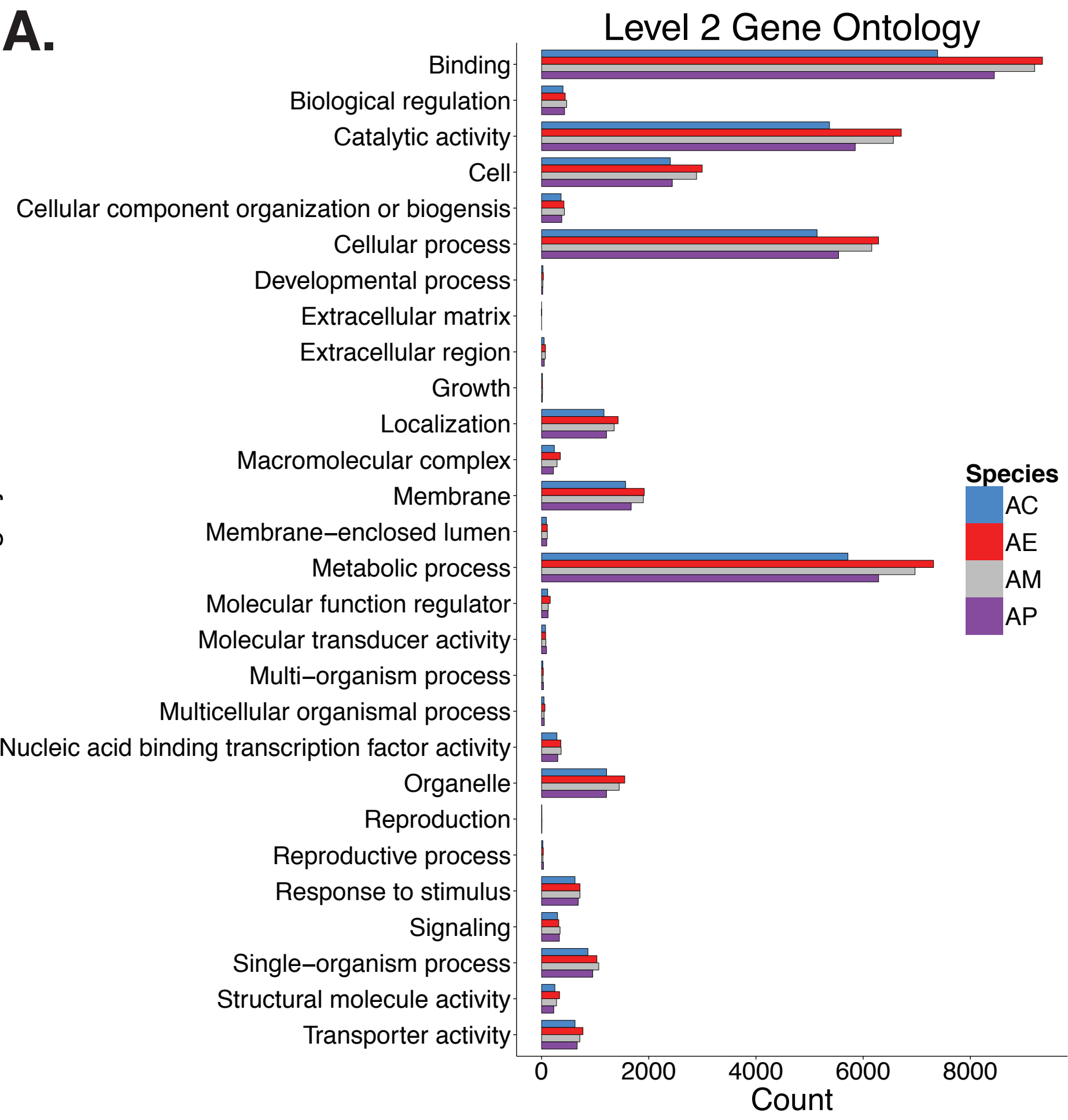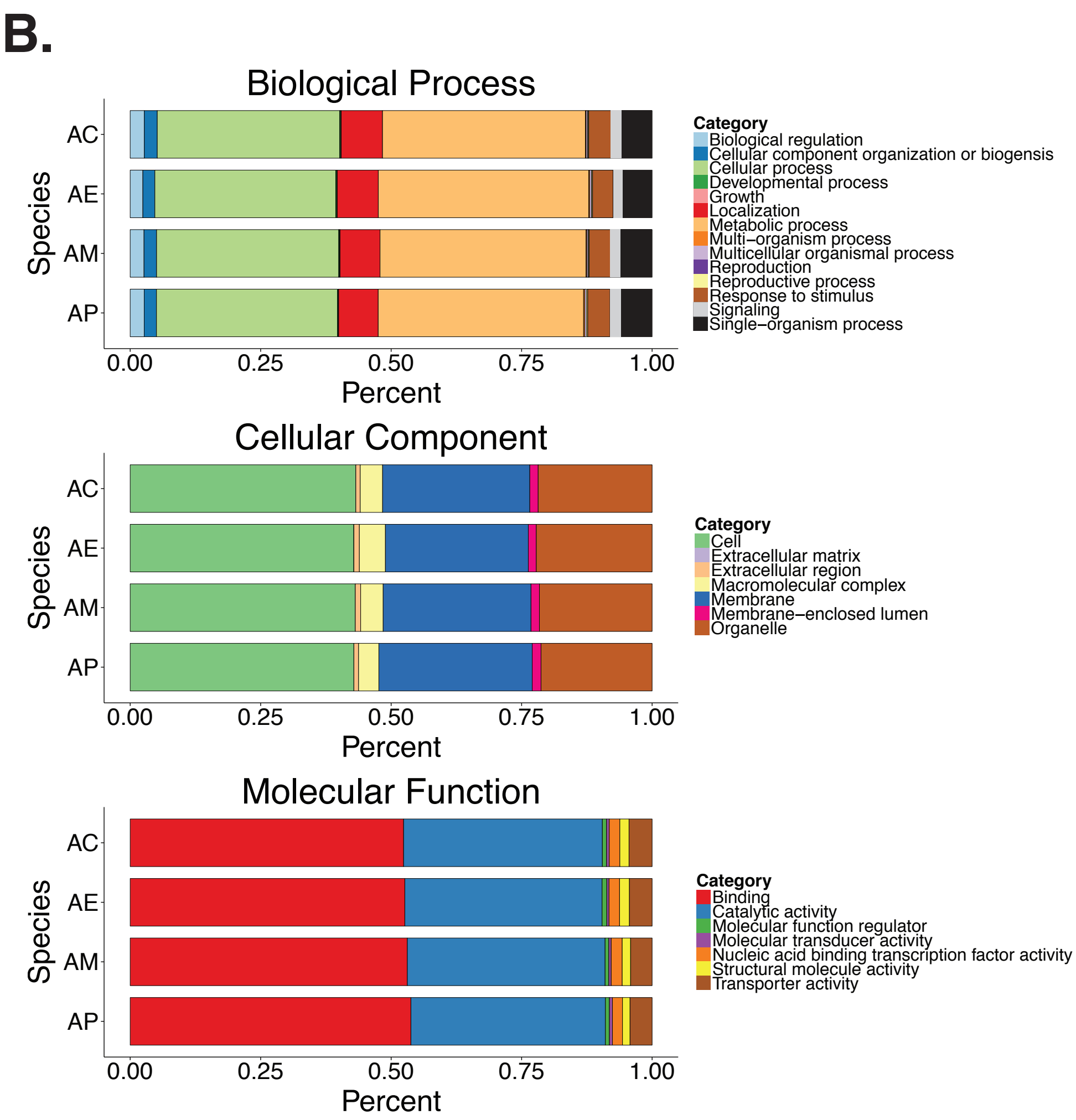

Abbreviations: AC, *A. cettoana*; AE, *A. erecta*; AM, *A. misera*; AP, *A. patens*.

Supplement: Additional file 3: Figure S1. — Counts and proportion of level 2 Gene Ontology annotations for Achimenes transcriptomes. (PDF 361 kb) [file 12864_2017_3623_MOESM3_ESM.pdf]

Additional file 10: Figure S6. Expression of genes involved in flower development in *Achimenes*.

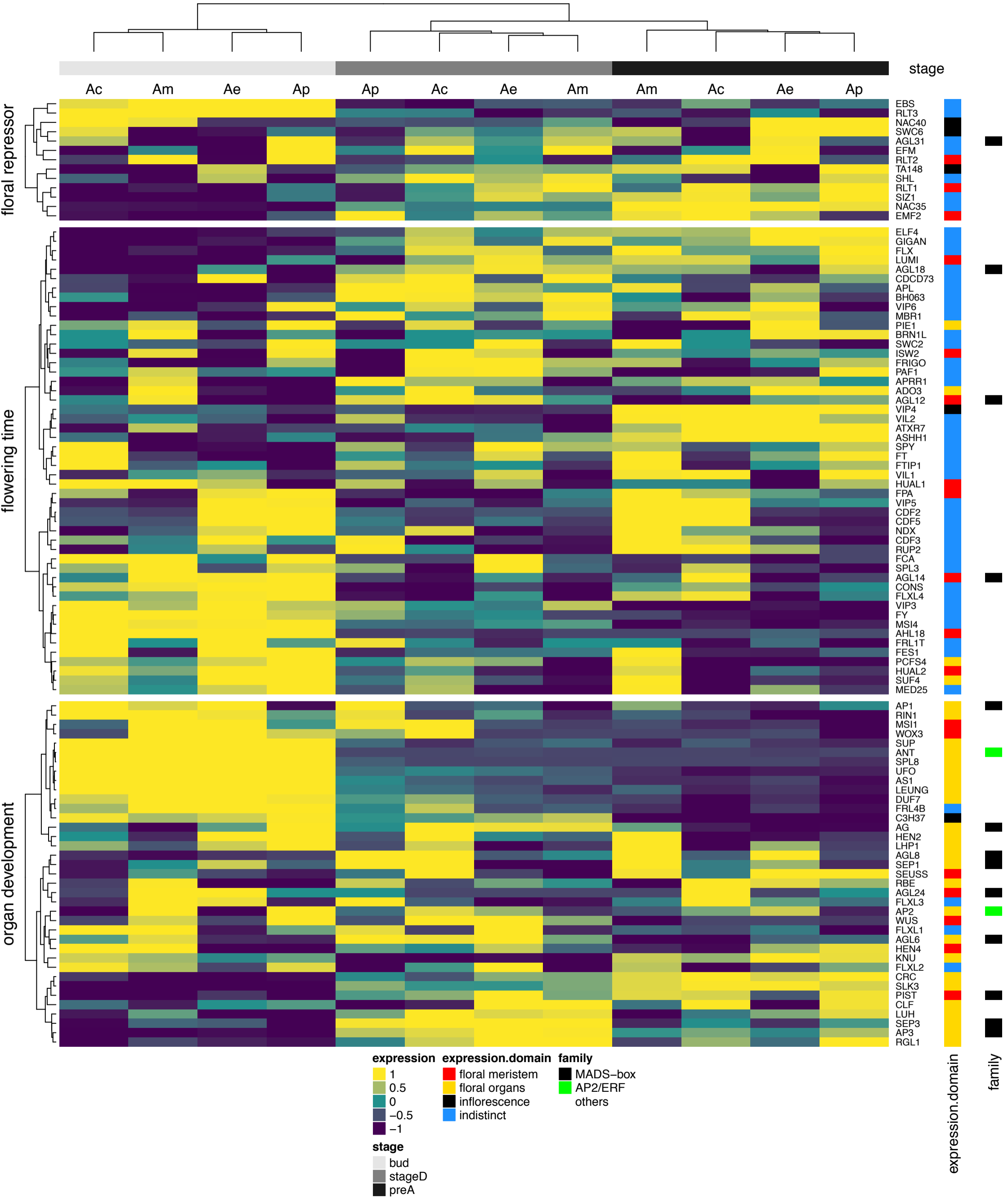

Supplement: Additional file 10: Figure S6. — Expression of genes involved in flower development in Achimenes. (PDF 346 kb) [file 12864_2017_3623_MOESM10_ESM.pdf]
