# Supplementary material for: Comparative transcriptome analyses of flower development in four species of Achimenes (Gesneriaceae)
Source: BMC Genomics. 2017 Mar 20;18:240. doi: 10.1186/s12864-017-3623-8 (PMC5359931; doi:10.1186/s12864-017-3623-8)

Additional file 6: Figure S3. Neighbor-joining tree of dihydroflavonol 4-reductase (*DFR*) homologs. Putative *Achimenes* DFR orthologs are highlighted in red. Bootstrap support >50 are indicated above branches.

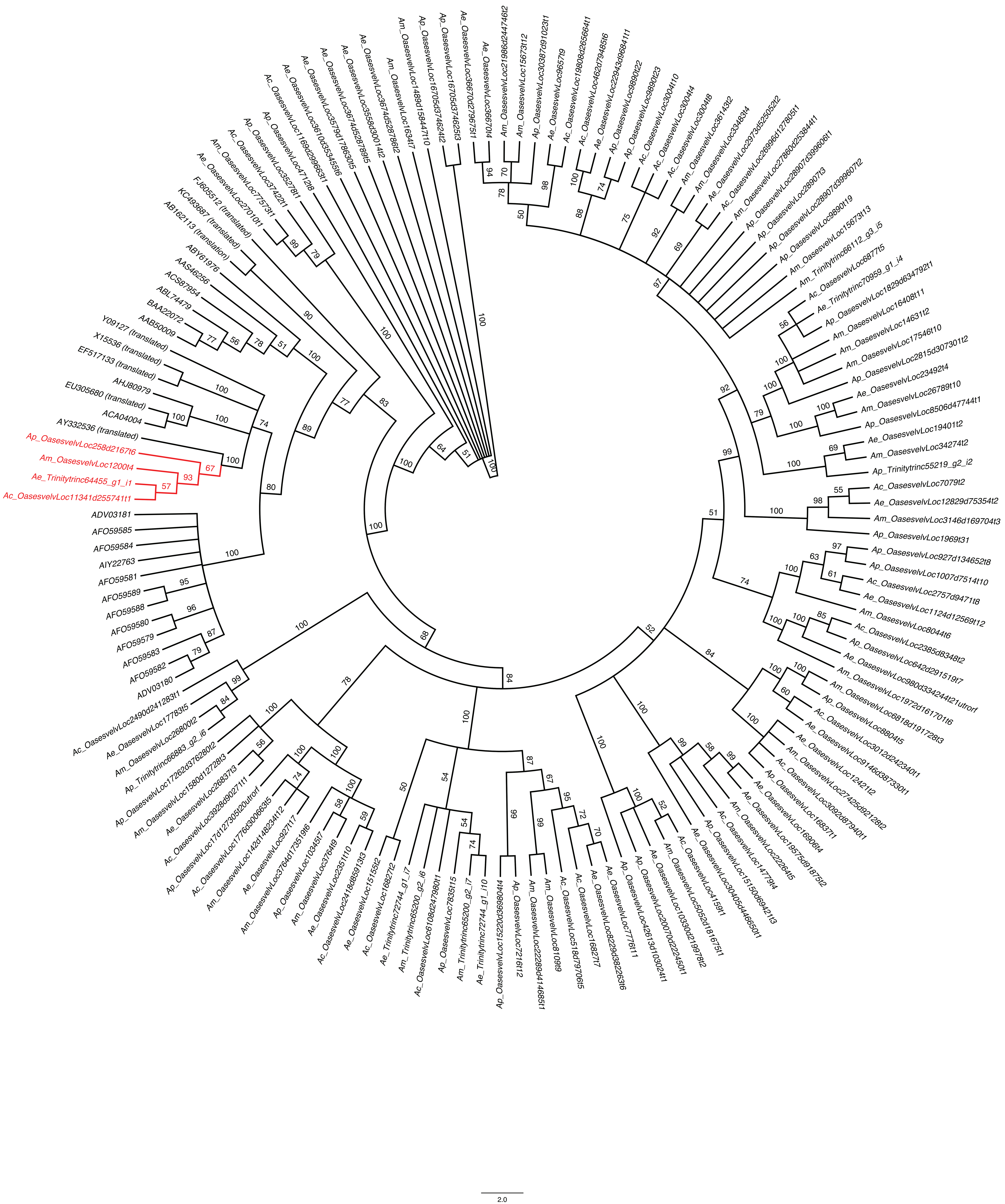

Supplement: Additional file 6: Figure S3. — Neighbor-joining tree of dihydroflavonol 4-reductase (DFR) gene family. Putative Achimenes DFR orthologs are highlighted in red. Boostrap support >50 are indicated above branches. (PDF 330 kb) [file 12864_2017_3623_MOESM6_ESM.pdf]
