# Supplementary material for: Comparative transcriptome analyses of flower development in four species of Achimenes (Gesneriaceae)
Source: BMC Genomics. 2017 Mar 20;18:240. doi: 10.1186/s12864-017-3623-8 (PMC5359931; doi:10.1186/s12864-017-3623-8)

Additional file 7: Figure S4. Neighbor-joining tree of flavonoid 3'-hydroxylase (***F3'H***) and flavonoid 3',5'-hydroxylase (***F3'5'H***) homologs. Putative *Achimenes* F3'H and F3'5'H orthologs are indicated in blue and red, respectively. Bootstrap support >50 are indicated above branches.

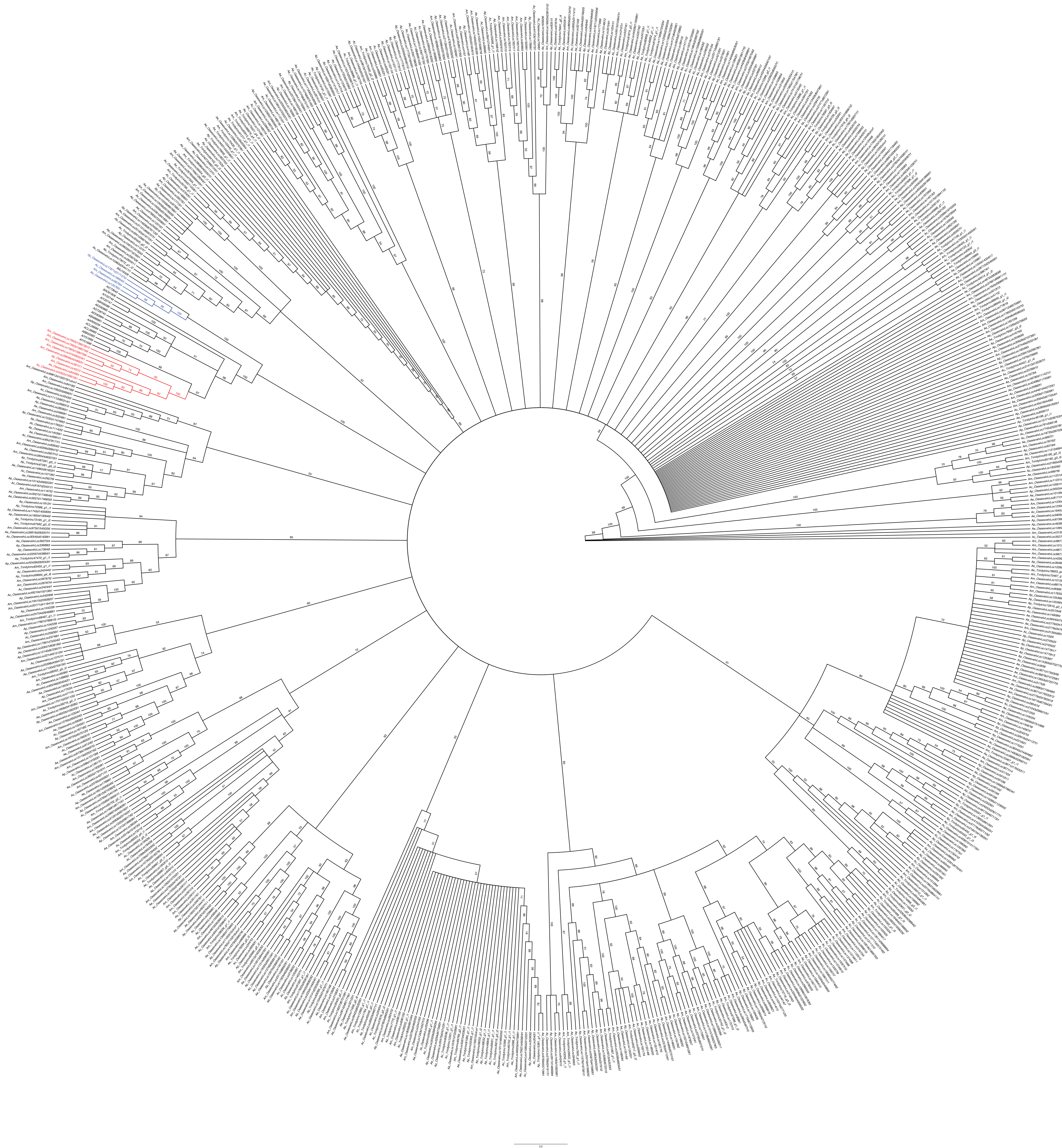

Supplement: Additional file 7: Figure S4. — Neighbor-joining tree of flavonoid 3′-hydroxylase (F3′H) and flavonoid 3′,5′-hydroxylase (F3′5′H) gene family. Putative Achimenes F3′H and F3′5′H orthologs are highlighted in red and blue, respectively. Bootstrap support >50 are indicated above branches. (PDF 1175 kb) [file 12864_2017_3623_MOESM7_ESM.pdf]
