# Supplementary material for: Comparative transcriptome analyses of flower development in four species of Achimenes (Gesneriaceae)
Source: BMC Genomics. 2017 Mar 20;18:240. doi: 10.1186/s12864-017-3623-8 (PMC5359931; doi:10.1186/s12864-017-3623-8)

Additional file 11: Figure S7. Neighbor-joining tree of R2R3-Mybs in Achimenes. Putative orthologs involved in regulating anthocyanin and carotenoid biosynthesis are highlighted in blue and orange, respectively. Bootstrap support >50 are indicated above branches.

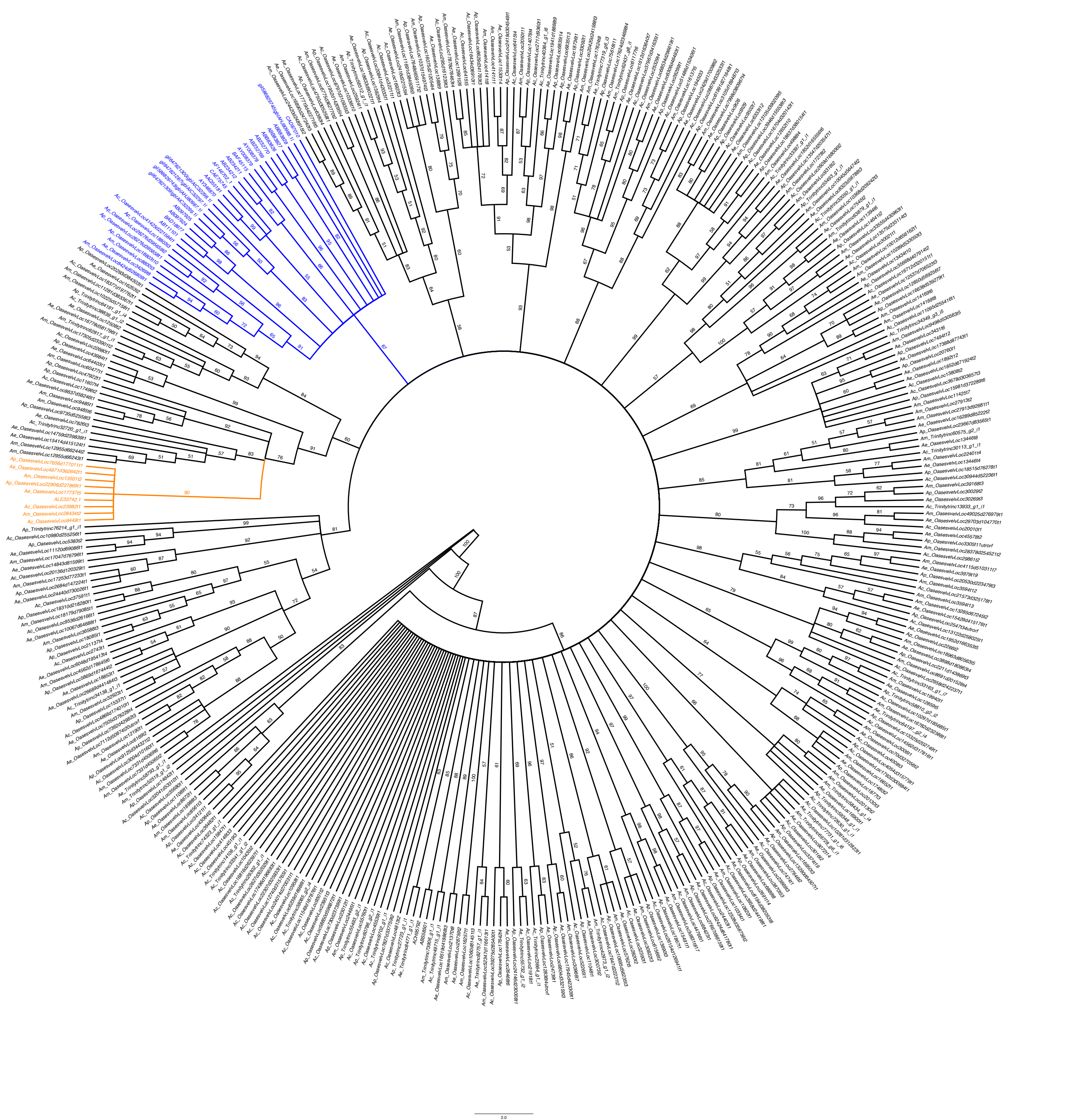

Supplement: Additional file 11: Figure S7. — Neighbor-joining tree of R2R3-Mybs in Achimenes. Putative orthologs involved in anthocyanin and carotenoid biosynthesis are highlighted in blue and orange, respectively. Bootstrap support >50 are indicated above branches. (PDF 921 kb) [file 12864_2017_3623_MOESM11_ESM.pdf]
