# Supplementary material for: Comparative transcriptome analyses of flower development in four species of Achimenes (Gesneriaceae)
Source: BMC Genomics. 2017 Mar 20;18:240. doi: 10.1186/s12864-017-3623-8 (PMC5359931; doi:10.1186/s12864-017-3623-8)

Additional file 15: Figure S8. Coexpression clusters for *Achimenes* determined using Poisson mixture models. Gene profiles are depicted as boxplots. Conditions are as follows: 1, Bud stage; 2, Stage D; and 3, Pre-Anthesis stage. A, *A. cettoana*; B, *A. erecta*; C, *A. misera*; D, *A. patens*.

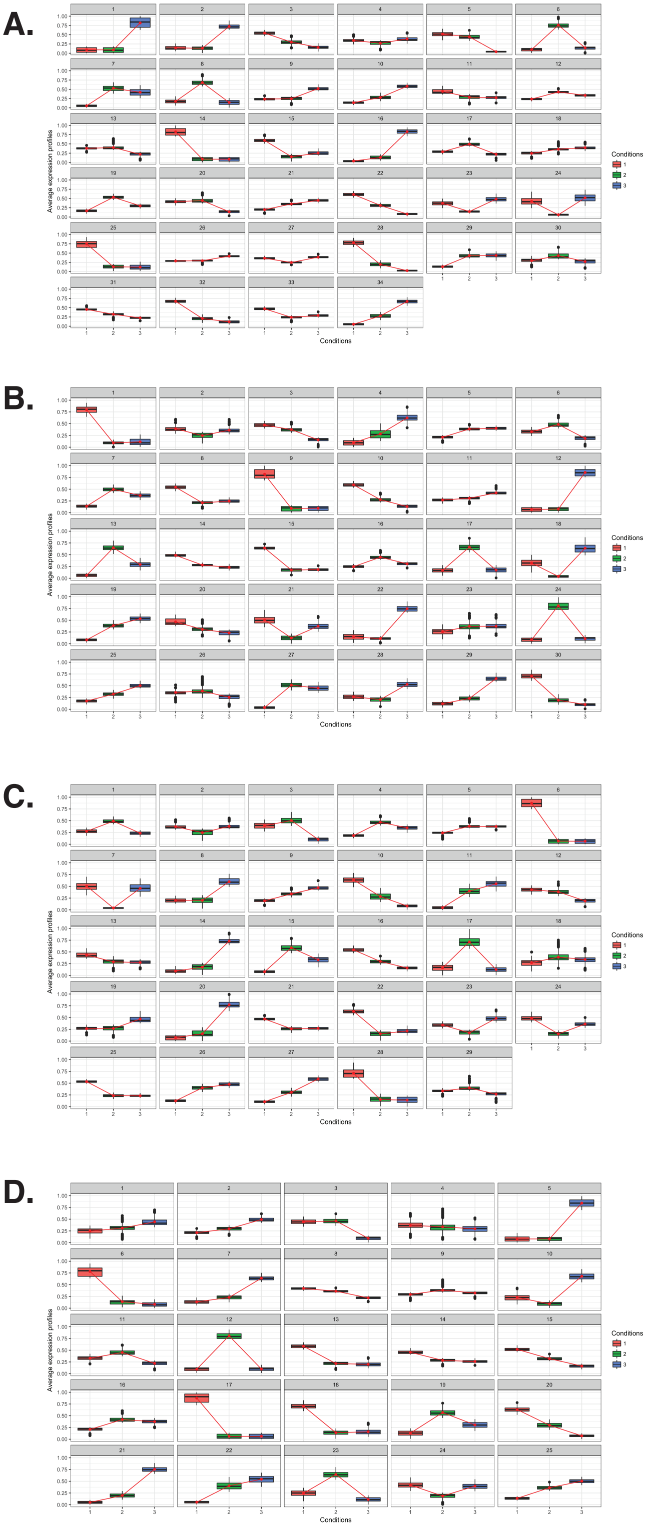

Supplement: Additional file 15: Figure S8. — Coexpression clusters for Achimenes determined using Poisson mixture models. Gene profiles are depicted as boxplots. Conditions are as follows: 1, Bud stage; 2, Stage D; and 3, Pre-Anthesis stage. A, A. cettoana; B, A. erecta; C, A. misera; D, A. patens. (PDF 4192 kb) [file 12864_2017_3623_MOESM15_ESM.pdf]
