# Supplementary material for: Comparative transcriptome analyses of flower development in four species of Achimenes (Gesneriaceae)
Source: BMC Genomics. 2017 Mar 20;18:240. doi: 10.1186/s12864-017-3623-8 (PMC5359931; doi:10.1186/s12864-017-3623-8)

Supplemental file 16: Figure S9. Maximum conditional probability of cluster membership assigned by coexpression clustering using Poisson mixture models. A, *Achimenes cettoana*; B, *A. erecta*; C, *A. misera*; D, *A. patens*.

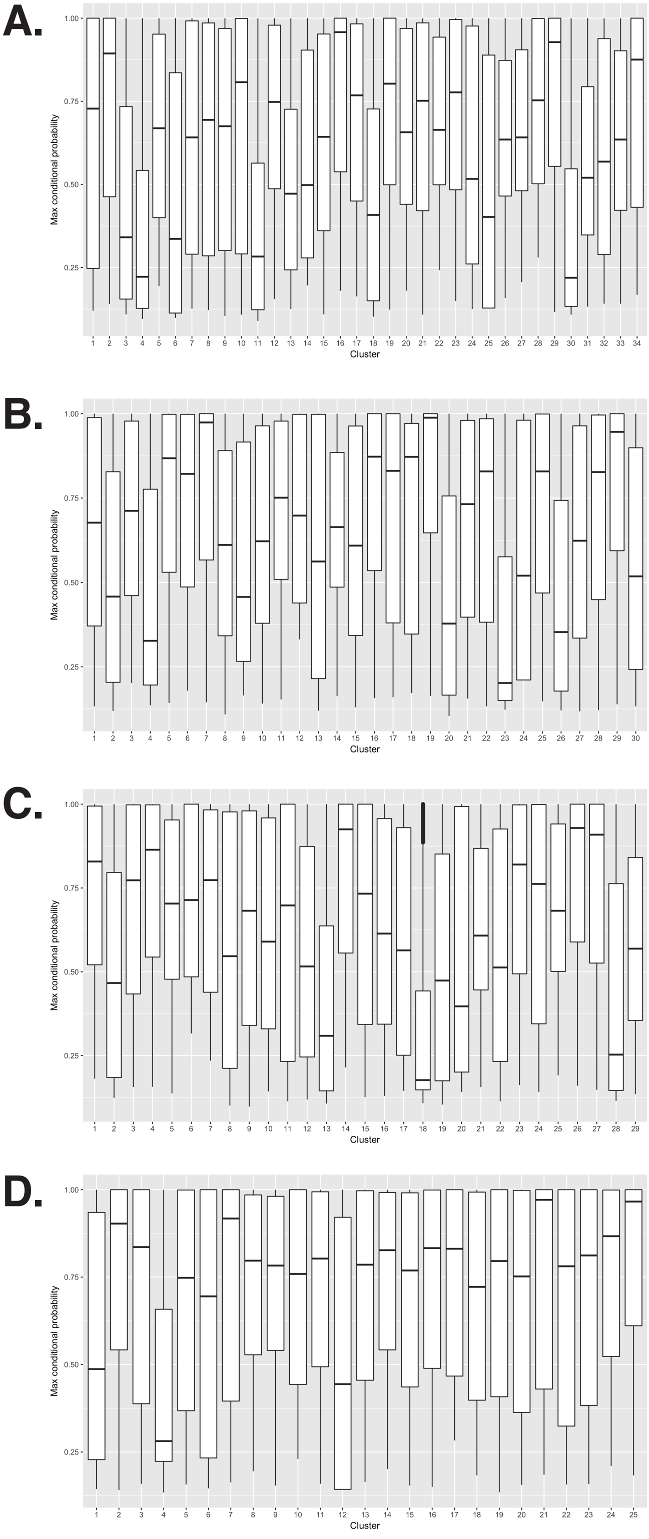

Supplement: Additional file 16: Figure S9. — Maximum conditional probability of cluster membership assigned by coexpression clustering using Poisson mixture models. A, Achimenes cettoana; B, A. erecta; C, A. misera; D, A. patens. (PDF 1176 kb) [file 12864_2017_3623_MOESM16_ESM.pdf]
